# Genetic architecture of cold tolerance in rice (*Oryza sativa*) determined through high resolution genome-wide analysis

**DOI:** 10.1371/journal.pone.0172133

**Published:** 2017-03-10

**Authors:** Ehsan Shakiba, Jeremy D. Edwards, Farman Jodari, Sara E. Duke, Angela M. Baldo, Pavel Korniliev, Susan R. McCouch, Georgia C. Eizenga

**Affiliations:** 1 University of Arkansas, Rice Research and Extension Center, Stuttgart, Arkansas, United States of America; 2 USDA/ARS Dale Bumpers National Rice Research Center, Stuttgart, Arkansas, United States of America; 3 Rice Experiment Station (RES), Biggs, California, United States of America; 4 USDA/ARS Plains Area, College Station, Texas, United States of America; 5 Department of Biological Statistics and Computational Biology, Cornell University, Ithaca, New York, United States of America; 6 School of Integrative Plant Sciences, Plant Breeding and Genetics section, Cornell University, Ithaca, New York, United States of America; National Institute for Plant Genome Research, INDIA

## Abstract

Cold temperature is an important abiotic stress which negatively affects morphological development and seed production in rice (*Oryza sativa* L.). At the seedling stage, cold stress causes poor germination, seedling injury and poor stand establishment; and at the reproductive stage cold decreases seed yield. The Rice Diversity Panel 1 (RDP1) is a global collection of over 400 *O*. *sativa* accessions representing the five major subpopulations from the *INDICA* and *JAPONICA* varietal groups, with a genotypic dataset consisting of 700,000 SNP markers. The objectives of this study were to evaluate the RDP1 accessions for the complex, quantitatively inherited cold tolerance traits at the germination and reproductive stages, and to conduct genome-wide association (GWA) mapping to identify SNPs and candidate genes associated with cold stress at these stages. GWA mapping of the germination index (calculated as percent germination in cold divided by warm treatment) revealed 42 quantitative trait loci (QTLs) associated with cold tolerance at the seedling stage, including 18 in the panel as a whole, seven in *temperate japonica*, six in *tropical japonica*, 14 in *JAPONICA*, and nine in *INDICA*, with five shared across all subpopulations. Twenty-two of these QTLs co-localized with 32 previously reported cold tolerance QTLs. GWA mapping of cold tolerance at the reproductive stage detected 29 QTLs, including seven associated with percent sterility, ten with seed weight per panicle, 14 with seed weight per plant and one region overlapping for two traits. Fifteen co-localized with previously reported QTLs for cold tolerance or yield components. Candidate gene ontology searches revealed these QTLs were associated with significant enrichment for genes related to with lipid metabolism, response to stimuli, response to biotic stimuli (suggesting cross-talk between biotic and abiotic stresses), and oxygen binding. Overall the *JAPONICA* accessions were more tolerant to cold stress than *INDICA* accessions.

## Introduction

Rice (*Oryza sativa* L.), a major cereal crop, thrives in both tropical and temperate regions around the world, including in the cool temperatures found at high elevations [1]. Based on phylogenic studies, rice cultivars are divided into two major varietal groups, *JAPONICA* which includes the *temperate japonica*, *tropical japonica*, and *aromatic* subpopulations, and *INDICA* which includes the *indica* and *aus* subpopulations [2, 3] [Note: upper-case letters are used to indicate the two major varietal groups, *INDICA* and *JAPONICA*, and lower-case letters to indicate subpopulations after McCouch et al. [4].]

The stress caused by low temperatures is a major limitation for rice production in temperate and subtropical zones [5], thus developing cold tolerant cultivars is a major focus of some rice breeding programs [1, 6, 7]. As a tropical species, the optimum temperature for germination and seedling growth in rice ranges from 25°C to 30°C. Cold stress occurs when the temperature falls below 17°C causing poor germination, seedling injury, poor stand establishment, and reduction of yield stability and productivity [1, 8, 9]. Despite the general sensitivity of rice to low temperatures, a range of cold tolerance exists among rice cultivars (accessions) [10, 11] and overall, *INDICA* accessions are reported to be more sensitive to cold stress than *JAPONICA* accessions [1, 3, 6, 12–14]. Identifying accessions with high levels of cold stress tolerance is an essential step toward developing cultivars with better cold tolerance and improving rice production in regions where cool temperatures limit rice yield.

Cold stress tolerance is important throughout the life cycle of the rice plant [8, 9], but especially in the early vegetative stages, i.e., at germination when the coleoptile elongates (S3 growth stage; [15]) and as the young seedling develops (V1 to V4 growth stages). The damage caused by low temperatures at the seedling stage is mainly observed as leaf rolling, necrosis, chlorosis and stunting [1, 10, 11]. When subjected to cold temperatures, seedlings demonstrate a wide range of genetic and physiological responses to protect their cell and plasma membranes, including activation of gene and protein expression, changes in membrane lipid composition, and accumulation of hydrophobic polypeptides [16–18].

At the early reproductive stages, especially at booting, when the panicle begins to emerge from the flag leaf sheath (R3 growth stage; [15]), the damage caused to the rice plants by cold stress is usually significantly greater than damage at the seedling stage [14] because it directly reduces yield. Seed yield is reduced because the cold temperature affects microspore development, such that fewer mature pollen grains develop in the anther, resulting in higher spikelet sterility [19, 20]. In fact, it has been shown that, reproductive stage-cold treatment at 15°C (day) and 10°C (night) increases spikelet sterility by up to 90% and reduced grain eating quality [21, 22].

Bi-parental mapping studies have identified 33 major QTLs distributed on all 12 chromosomes (chr.) associated with cold tolerance at the germination and seedling stages [6, 23–25], as well as, at the booting stage [6, 24, 26]. The large number of QTLs identified suggests that cold tolerance in rice expressed at both germination and booting is quantitatively inherited and controlled by many genes. In these bi-parental populations, the majority of QTLs associated with cold tolerance were derived from a *JAPONICA* parent. For example, in the RIL (recombinant inbred line) population derived from M-202, a cold tolerant *temperate japonica* crossed with IR50, a cold sensitive *indica*, QTLs associated with cold tolerance at the vegetative stages were located on seven chromosomes [1], while QTLs associated with reproductive stage-tolerance were found on eight chromosomes [6]. Further genetic analysis of this population revealed two linked candidate genes, *OsGSTZ1* and *OsGSTZ2*, underlying a QTL for cold tolerance at the seedling stage on chr. 12 [10]. Additional cold tolerance QTL have been discovered in bi-parental populations at the seedling stage [25] and at the booting stage [24], and in advanced interconnected breeding (IB) lines at the booting stage [27].

Genome wide association (GWA) mapping is a powerful approach for identifying QTL using collections of unrelated (or distantly related) individuals or cultivars [28]. Initially this approach was used to identify QTLs associated with chilling tolerance in crops like maize and barley [29, 30] but more recently GWA mapping has been used to understand the genetic variation of cold tolerance in rice [13, 14, 31, 32]. Using a mini-core collection of 174 Chinese rice accessions and 273 SSR markers, Pan et al. [14] investigated cold tolerance at the seedling and booting stages based on seedling survival and seed setting, respectively. Overall *JAPONICA* was more tolerant than *INDICA* for all stages. The GWA mapping detected 51 QTL associated with cold tolerance dispersed across all 12 chromosomes with 22 QTL associated with the seedling stage and 33 QTL with the booting stage. GWA mapping of the Hokkaido Rice Core Panel, a collection of 63 Japanese rice landraces and breeding lines which was genotyped with 117 markers, revealed six QTL associated with cold tolerance at heading and 17 QTL associated with low temperature germinability [31]. Lv et al. [13] evaluated a large collection of 529 rice accessions, including 202 accessions from the China Core Collection and 327 from the World Core Collection, under natural chilling and cold shock stress conditions at the seedling stage. Subsequent, GWA studies using more than 4 million SNPs identified 132 loci associated with at least one of the 16 traits evaluated to measure these two cold stress conditions. Haplotype analysis of these accessions for the *OsMYB2* gene involved with cold tolerance, revealed the *INDICA-JAPONICA* differentiation, with *JAPONICA* being more cold tolerant and having a wider latitudinal distribution.

The Rice Diversity Panel 1 (RDP1) is a global collection of over 400 rice accessions representing the five major subpopulations found in the *INDICA* and *JAPONICA* varietal groups. Wang et al. [32] evaluated 295 RDP1 accessions genotyped with 36,901 high quality SNPs for cold tolerance at the seeding stage. Subsequent, GWA mapping identified 67 QTL located on 11 chromosomes. Recently, the RDP1 collection was genotyped with 700,000 SNP markers using a high density rice array (HDRA) [4] and this publicly available genotyping dataset was used for GWA mapping at the germination and reproductive stages. The objectives of this study were to (a) evaluate the RDP1 accessions for cold tolerance at the germination and reproductive stages, (b) conduct GWA mapping using the HDRA SNP genotypes and the suite of bioinformatics tools developed for the RDP1, and (c) identify SNPs and underlying candidate genes associated with tolerance to cold stress in rice at these critical developmental stages.

## Materials and methods

### Plant materials

Seed of 421 accessions included in the RDP1 [33] were obtained from the USDA/ARS Genetics Stocks-*Oryza* (GSOR) collection with 87% of the seed used in this test being produced in the field during the 2011 growing season and stored at 4°C after harvest until being prepared for this study. (Only three RDP1 accessions were not included in this evaluation, GSOR312003, GSOR312016 and GSOR312019.) The RDP1 accessions in this study included 161 *INDICA* accessions (94 *indica*, 60 *aus* and seven admixed), 250 *JAPONICA* accessions (100 *tropical japonica*, 101 *temperate japonica*, 15 *aromatic* (Group V) and 34 admixed), and 10 *INDICA-JAPONICA* admixed accessions (10 with ancestry in both *INDICA* and *JAPONICA*). A cut-off of 70% ancestry was used to classify the accessions into one of the five subpopulation groups or into one of the three admixture classes: admixed *INDICA* (*aus-indica*), admixed *JAPONICA* (*tropical japonica-temperate japonica-aromatic*) or admixed *INDICA-JAPONICA* [4]. For 23 accessions which did not have HDRA genotypes, thus not classified by McCouch et al. [4], the classification was based on SSR marker data [33]. [Subpopulation information is summarized in an Excel file which can be downloaded at the GSOR website (https://www.ars.usda.gov/southeast-area/stuttgart-ar/dale-bumpers-national-rice-research-center/docs/rice-diversity-panel-1/).]

Controls for the evaluation of cold tolerance at the germination stage included Quilla66304 (PI 560281) as highly cold tolerant, Lemont (PI 475833) as moderately cold tolerant and Zhe733 (GSOR 100355; PI 634573 MAP), as cold-sensitive (low cold tolerance). Controls for evaluating cold tolerance at the reproductive stage included Mustakilik from Uzbekistan, Jinbu from South Korea, and Calmochi-101 (PI 494104), Calmati-201 (PI 608665), M-205 (PI 615535) and L-206 (PI 645472), all developed for production in the temperate USA environment in California.

### Evaluation of cold tolerance at germination

Cold tolerance at germination was evaluated using the “ragdoll method” developed at the Dale Bumpers National Rice Research Center near Stuttgart, Arkansas. To conduct this evaluation 20 grams of seed were inspected with a “light box” which has an incandescent light fixture mounted in a wooden box, with a hole cut in the top. Seed were placed in a petri dish, placed over the hole and light shown through the dish so that opaque seed, many of which were the damaged, diseased or broken seed, could be identified and removed. This resulted in fully developed, viable, translucent seed being selected for the study. The seed sample was sterilized for ten minutes in 15 ml of one part bleach (5.25% sodium hypochlorite) to one part water solution and then rinsed three times with 45 ml of sterile deionized water. Underdeveloped seed floated to the surface during these rinsing steps and were removed. For each accession, five “ragdolls” were prepared by placing 30 well-developed seeds on a sterilized industrial strength, triple fold, paper towel and rolling up the paper towel to make a “ragdoll”. Ten ragdolls, including the three control varieties and seven RDP1 accessions, were placed on each tray in a random order with each tray representing one replication. The ragdolls in the tray were wet with 100 ml of a fungicide solution (150 ul Dynasty^®^: 1 L deionized water). The tray was covered with plastic wrap to decrease evaporation and covered with a second tray. Four binder clips, one on each side, were used to hold the trays together. The accessions were arranged in a randomized complete design with three cold replications (each accession occurred on three separate trays) and two warm replications. Three trays were randomly placed in a dark ‘cold’ incubator set at 12°C for 35 days and two trays were placed in a dark ‘warm’ incubator set at 30°C for five days to correct for the percentage of germination (i.e. viability) of a given accession. After the appropriate time period, the trays were removed from the cold or warm treatment and each seed was classified into one of three categories based on coleoptile length: the seeds classified as having “high tolerance” had coleoptiles longer than 5 mm, those classified as having “low tolerance” had coleoptile lengths less than 5 mm long, and those which had no coleoptile elongation were classified as not germinating and had no cold tolerance. Seed which had fungal contamination were removed from the analysis.

Germination rates of check varieties were evaluated across all trays within the warm and cold germination trials by evaluating process control variability charts, Analysis of Mean (ANOM) and simple regression across the 61 trays [34, 35]. There were no trends detected in germination in either the warm or cold trials across the 61 sets defined as three replications in cold treatment and two in warm; therefore, no adjustments were necessary for the test accessions. To determine the most reliable phenotype estimate to use for association mapping, we evaluated both means and adjusted means using six different calculations based on the coleoptiles being more than 5 mm long, coleoptile emergence (combining coleoptiles more than 5 mm long and less than 5 mm), and correcting for germination under the warm temperature using the means procedure [34]. Based on the Q-Q plots and lambda values obtained as part of the GWA analyses described below, we determined the best measure of cold tolerance at germination was the mean adjusted for germination under warm temperatures. This was calculated as the ratio of the percentage of seeds with coleoptiles >5 mm in the cold treatment divided by the percentage of the seeds with coleoptile length >5 mm in the warm treatment, hereafter described as the “germination index”.

### Evaluation of cold tolerance at the reproductive stage

For this study, 227 RDP1 *JAPONICA* accessions, excluding the *aromatic* (Group V) accessions, were planted on April 21, 2014 in a greenhouse modified to evaluate cold tolerance at the reproductive stage located at the California Cooperative Rice Research Foundation near Biggs, California. At the time of planting, the day length was 13.5 hrs. Seeds from each accession were planted in two liter pots and each pot was randomly placed on one of two benches with each bench being considered as one replication. The plants were fertilized with nitrogen (47 kg ha^-1^) and potassium (27 kg ha^-1^) at 14 days and 40 days after planting. At 45 days after planting, when the panicle initiation typically begins in the earliest adapted varieties (daylength = 14.75 hrs), the night time temperature was adjusted to 12°C between midnight (00:00 h) and 07:00 h. The average day time temperature was 27.3°C during this time. The cold night time treatment continued for several weeks until 186 accessions reached the booting stage. (The remaining 41 accessions did not mature, in part due to photoperiod sensitivity, and thus no data were collected.) The days to 50 percent heading, days to harvest maturity, plant height, percent panicle sterility, number of panicles per plant, and seed weight per plant were recorded. Percent panicle sterility was scored as follows: category “1” is 0 to 5% sterile florets, “2” is 6–12%, “3” is 13–18%, “4” is 19 to 40%, “5” is 41 to 60%, “6” is 61 to 90%, and “7” is 91 to 100%.

The procedure ANOVA (Analysis of Variance) in SAS [34] using the controls revealed no effect due to placement on one of the two greenhouse benches used for this experiment. There was no significant correlation between days to heading and the other three traits measured, thus it was excluded from the GWA analysis. Based on the Q-Q plots (described below), the means identified for further evaluation were percent sterility, mean of seed weight per plant (SWPlt) and mean seed weight per panicle (SWPan).

### Genome-wide association analysis

GWA studies were run using the analysis pipeline and HDRA dataset consisting of 700,000 SNPs described by McCouch et al. [4]. In the pipeline, principal components (PCs) were calculated from the genetic correlation matrix using the svd function of the R statistical package [36]. The SNP data were filtered to include only SNPs with a minor allele frequency (MAF) > 0.05 in each subpopulation. Efficient Mixed-Model Association eXpedited (EMMAX vs beta-07Mar2010) was used to calculate a linear mixed model that adjusts for population structure by including a kinship matrix covariate using the identity by descent (IBS) method of EMMAX [37]. Three PCs were included in the mixed model when analyzing across all subpopulations, but not when analyzing individual subpopulations. The quantile-quantile (Q-Q) plots (S3 Fig) for each group were produced to visualize the distribution of the test statistics and evaluate the inflation factor, lambda (λ). Based on the approximate significance value where the observed number of p-values exceeds the expected number in the Q-Q plots, a significance threshold of 10^−4^ was used across all analyses. The GWA analysis pipeline scripts and SNP data are available at www.ricediversity.org. GWA was run for the three subpopulation groups, *indica*, *tropical japonica* and *temperate japonica*, as well as, for the two varietal groups, *INDICA* (*aus*, *indica* and admixed *aus-indica*) and *JAPONICA* (*temperate japonica*, *tropical japonica* and admixed *temperate-tropical-japonica*) as described in McCouch et al. [4]. The *aus* and *aromatic* subpopulations did not have enough accessions to conduct a separate GWA analysis of the subpopulation group. QTL regions were identified as having three or more SNPs above the threshold level. Before assigning a QTL to a region, we required three SNPs below the critical p-value because rice (as a primarily inbreeding species) has a gradual decay of LD, especially compared to outcrossing species such as maize. Given the high density SNP coverage we would expect it to be rare for an isolated significant SNP to be detected within a genomic region. By requiring at least three SNPs having significant p-values in a 150–200 kb region, we hoped to reduce the number of false positives. This method of selecting at least three SNPs in a 150–200 kb region was also used by McCouch et al. [4]. All significant SNPs identified are listed in S1 Table by trait and subpopulation group.

As background, the Q-Q plots and Manhattan plots from the GWA mapping were examined based on five different calculations for the cold tolerance, some of which included the germinated seed with coleoptile lengths less than 0.5 mm. The GWA results which included the coleoptile length less than 0.5 mm had extremely low values for lambda (significant deviation from expected), thus very few significant SNPs were identified, with most below the threshold p-value. This made it difficult to analyze the GWA mapping data. Considering that our long term objective is to identify cold tolerant germplasm for incorporation into rice breeding programs, selecting those which had the best germination under cold conditions is important, thus we chose to use only those that had coleoptile lengths greater than 0.5 mm to calculate the germination index.

### Linkage Decay (LD) estimation

Pairwise linkage between adjacent sites across the genome was estimated for SNPs in each subspecies and each subpopulation using the 2.6 million SNPs publicly available from the RGP 3K project [38]. Tassel software v5.2.4 [39] was run with the following parameters: " -ldPermNum 100000 -ldRapidanalysis false". Local pairwise LD between adjacent SNPs was visualized alongside the MSU7 genome assembly, HDRA SNPs, RGP 3K SNPs, MSU7 gene and repeat annotations, and annotations downloaded from the Gramene database[40] using the genome viewer JBrowse v1.11.5[41] instance on Ricebase[42]. The pairwise LD between SNPs was used as a rough visual approximation of the local rate of LD decay to guide manual selection of candidate genes. The “GWAS Viewer” tool (http://ricediversity.org)[4] was used to provide zoomed in views of SNP significance vs chromosome location in QTL regions relative to candidate genes.

### Gene Ontology (GO) analysis

Gene ontology (GO) term enrichment analysis was performed using the list of candidate genes derived from the cold-tolerance GWA mapping at the germination stage. Gene IDs were supplied to the agriGO analysis toolkit [43] using the Fisher statistical method to detect significantly enriched ontology terms.

## Results

### Analysis Of Variance (ANOVA) at the germination stage

**Fig 1:**
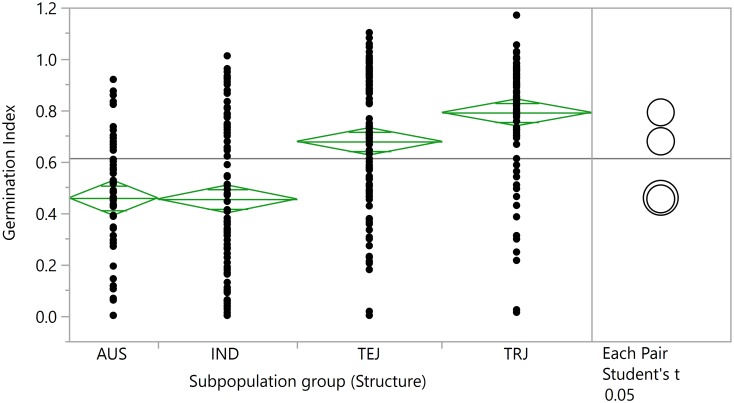
Distribution of the Rice Diversity Panel 1 accessions by subpopulation group for cold tolerance at germination. Cold tolerance described as the “Germination Index” was calculated as the ratio of the mean percentage of seeds with coleoptiles >5 mm under cold treatment to the mean percentage of seeds with coleoptiles >5mm under warm treatment. The *temperate japonica* and *tropical japonica* accessions were significantly more tolerant than accessions classified as *indica* and *aus*, the *INDICA* subspecies. Based on the Student’s t-test (p<0.05) there were no differences between *indica* and *aus* subpopulations.

As summarized in Fig 1 and Table 1, *JAPONICA* accessions were more tolerant to cold stress at germination than *INDICA* accessions, based on mean germination index. A wide range of variation was observed within each subpopulation (Fig 1; S2 Fig), but the mean germination index of *tropical japonica* accessions showed them to be more cold tolerant than *temperate japonica* accessions, and both were more tolerant than either *indica* or *aus* (Student’s t-test, p<0.05). Of the 421 RDP1 accessions evaluated in this study, 19.7% (83 accessions) had a higher germination index than Quilla66304 [(≥0.89+0.02 (SE)], the cold tolerant control (S2 Table; S1 Fig).

**Table 1:** Summary statistics for cold tolerance at the germination and reproductive stages. The mean, range and standard error are given for the germination index (calculated as percent germination in cold divided by warm treatment) based on the two subspecies, *INDICA* and *JAPONICA*, and four subpopulations *aus*, *indica*, *temperate japonica* and *tropical japonica*. At the reproductive stage, 186 *JAPONICA* accessions which produced panicles were evaluate for three traits.

Group\Trait	No. accessions	Mean	Range	SE^1)^
**Germination**				
*ALL*	421	0.63	0.00–2.67	0.02
*INDICA*	161	0.46	0.00–1.01	0.02
*aus*	60	0.46	0.00–0.92	0.03
*indica*	94	0.45	0.00–1.01	0.03
*JAPONICA*	250	0.74	0.00–2.67	0.02
*temperate japonica*	100	0.71	0.00–2.67	0.03
*tropical japonica*	101	0.79	0.01–1.17	0.02
**Reproductive**				
Percent sterility ^2)^	186	4.0	1.5–7.0	0.1
Seed weight per plant	186	5.00	0.10–20.45	0.28
Seed weight per panicle	186	0.71	0.01–2.56	0.03

^1)^ SE: standard error (standard deviation/square root no. accessions)

^2)^ Percent sterility was rated with the following categories: category 1 (0 to 5% sterility), 2 (6 to 12%), 3 (13 to 18%), 4 (19 to 40%), 5 (41 to 60%), 6 (61 to 90%) and 7 (91 to 100%).

### Analysis of variance at reproductive stage

RDP1 *JAPONICA* accessions (n = 227) were evaluated for cold stress at the reproductive stage in the greenhouse but only 186 produced panicles during the time period allotted for this study. Only *JAPONICA* varieties were evaluated because they were significantly more tolerant to cold stress at germination than *INDICA* accessions, and greenhouse space was limited. A wide range of variation was observed for all three traits, percent sterility, SWPan and SWPlt (Table 1; S2 Fig; S3 Table). The *JAPONICA* accession means for each trait were compared to Mustakilik, the most reproductively cold tolerant control variety. Those accessions which rated one standard error above Mustakilik included 64 accessions with less sterility, 15 accessions having more SWPlt and 16 having more SWPan (S3 Table). Of these accessions, only seven were identified as cold tolerant for all three reproductive traits.

Comparing results from the germination and reproductive stage screens, 20 of these *JAPONICA* accessions, which rated as cold tolerant for at least one reproductive trait, exhibited cold tolerance at germination (S3 Table). Surprisingly, of the seven accessions identified as tolerant for all three reproductive traits, only Karabaschak and Rikuto Norin 21 were identified as highly cold tolerant based on their germination index (S2 Table).

### Genome-wide association analysis for cold tolerance at the germination stage

**Fig 2:**
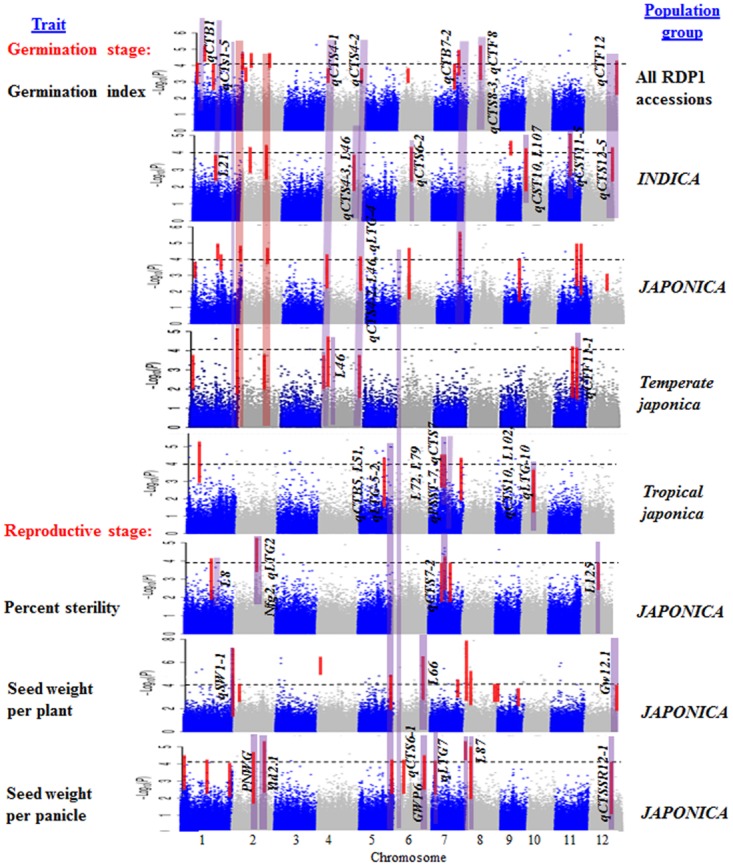
Manhattan plot from GWA analyses associated with cold tolerance at germination and the reproductive stages. The *x*-axis represents SNP positions across the entire rice genome by chromosome and the *y*-axis is the negative logarithm *p-*value of each SNP. The dotted line is the threshold (*p*-value > 10^−4^) and SNPs above this threshold were identified as significant. The red band represents candidate genes across each group which were associated with significant SNPs within haplotype blocks and the purple band the location of previously reported QTLs associated with cold tolerance or, in the case of the reproductive traits, seed yield.

GWA mapping for the germination index was run using the entire RDP1 panel (n = 421), and separately for each varietal group and each subpopulation. We did not analyze the *aus* subpopulations alone because of its small sample size. GWA analysis of the *indica* subpopulation revealed no group of three or more significant SNP associations, thus the results are not presented. Manhattan plots were generated for each group to illustrate the significance of SNP associations in the GWA analysis (Fig 2). In each group, SNPs were detected above the significance threshold (p-value = 0.0001) and QTL regions were identified when 3 or more significant SNPs occurred within a 1 Mb interval.

**Fig 3:**
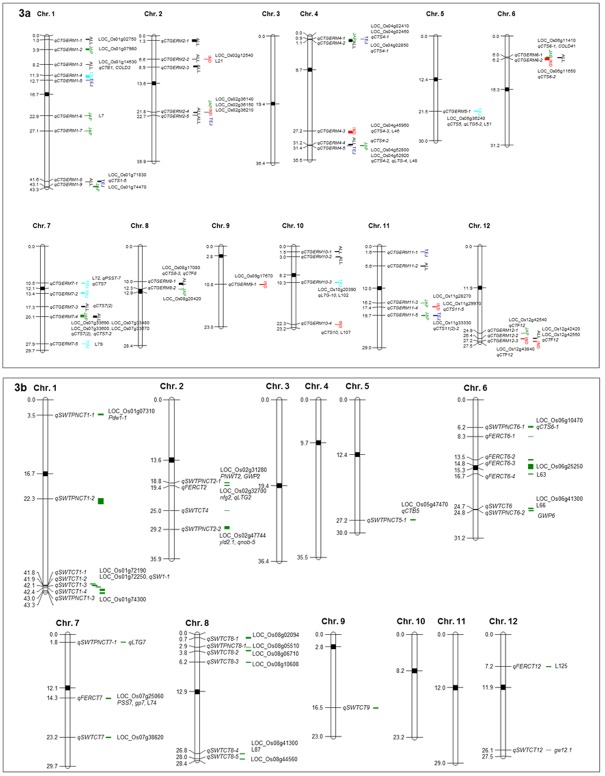
Location of the QTLs identified by the GWA mapping for cold tolerance at the germination stage (Fig 3a) and reproductive stage (Fig 3b), including co-located QTL and cadidate genes that were previously reported. The position of the QTL regions correspond to Table 2 for the germination stage (Fig 3a) and Table 3 for the reproductive stage (Fig 3b). The subpopulation group(s) where the QTL was identified are listed as *ALL* (all RDP1 accessions-black), IND (*INDICA*-red), JAP (*JAPONICA*-green), TEJ (*temperate japonica*-dark blue) and TRJ (*tropical japonica*-light blue). The “L” is for Locus [13]. Each significant SNP located in a particular QTL region is identified by a bar, thus the wider bars have more SNPs associated with the particular QTL.

GWA mapping identified a total of 18 QTL in the RDP1 as a whole (*ALL*) associated with cold tolerance (measured by germination index), including nine in *INDICA* (of which four were co-located with those identified in *ALL*), 13 in *JAPONICA*, (of which three were co-located with *ALL* and one was co-located with *INDICA*), seven in *temperate japonica*, and six in *tropical japonica* (Table 2; Fig 3a).

**Table 2:** Summary of SNP positions for cold tolerance at germination as identified by GWA mapping. All SNPs above the threshold (p-value = 0.0001) are included. The accessions were grouped as *ALL* RDP1 accessions, *INDICA*, *JAPONICA*, *temperate japonica* and *tropical japonica*. Reported QTLs and candidate genes associated with cold tolerance which are co-located to these segments are identified.

QTL	Population group ^1)^	Chromo-some	Position of segment (Mb)	SNP position (Mb)	p-value	No. significant SNPs	Co-located QTL ^2)^ ^3)^	Co-located candidate gene ^4)^
*qCTGERM1-1*	*ALL*	1	0.939–1.066	0.956	3.00E-04	25		LOC_Os01g02750
*qCTGERM1-2*	*JAPONICA*	1	3.712–3.995	3.860	3.55E-04	7		LOC_Os01g07980
*qCTGERM1-3*	*ALL*	1	8.161–8.228	8.190	4.23E-05	6	*qCTB1* ^5)^, *COLD3*	LOC_Os01g14630
*qCTGERM1-4*	*tropical japonica*	1	11.338–11.881	11.338	5.35E-06	3		
*qCTGERM1-5*	*temperate japonica*	1	12.710–12.725	12.714	4.69E-04	3		
*qCTGERM1-6*	*JAPONICA*	1	22.892–22.917	22.920	5.13E-05	3	L7 ^6)^	
*qCTGERM1-7*	*JAPONICA*	1	27.060–27.169	27.060	4.66E-04	3		
*qCTGERM1-8*	*ALL*	1	41.518–41.786	41.603	8.79E-05	26	*qCTS1-5*	LOC_Os01g71830
	*temperate japonica*	1	41.750–41.769	41.757	7.07E-06	5	*qCTS1-5*	
*qCTGERM1-9*	*JAPONICA*	1	43.039–43.223	43.128	2.29E-04	5		LOC_Os01g74470
*qCTGERM2-1*	*ALL*	2	1.068–1.655	1.329	1.66E-04	8		
*qCTGERM2-2*	*ALL*	2	6.557–6.624	6.557	2.99E-05	4	L21 ^6)^	LOC_Os02g12540
	*INDICA*	2	6.557–6.561	6.557	7.21E-05	4	L21 ^6)^	LOC_Os02g12540
*qCTGERM2-3*	*ALL*	2	8.487–8.905	8.885	2.25E-04	5		
*qCTGERM2-4*	*ALL*	2	21.772–21.872	21.797	7.65E-05	7		LOC_Os02g36150
	*INDICA*	2	21.810–21.839	21.838	1.71E-04	4		LOC_Os02g36140, LOC_Os02g36210
	*JAPONICA*	2	21.772–21.808	21.797	1.16E-04	5		LOC_Os02g36150
	*temperate japonica*	2	21.772–21.812	21.808	1.66E-04	13		LOC_Os02g36150
*qCTGERM2-5*	*ALL*	2	22.650–22.661	22.661	1.39E-05	4		
*qCTGERM4-1*	*JAPONICA*	4	0.866–1.116	0.866	2.46E-04	7	*qCTS4-1*	LOC_Os04g02450
	*temperate japonica*	4	0.866–0.894	0.866	9.33E-04	3	*qCTS4-1*	LOC_Os04g02410
*qCTGERM4-2*	*ALL*	4	1.103–1.648	1.116	5.63E-05	4	*qCTS4-1*	LOC_Os04g02850
*qCTGERM4-3*	*INDICA*	4	27.214–27.766	27.214	3.54E-04	36	*qCTS4-3*, L46 ^6)^	LOC_Os04g45950
*qCTGERM4-4*	*ALL*	4	31.047–31.230	31.159	3.78E-04	8	*qCTS4-2*	
*qCTGERM4-5*	*JAPONICA*	4	31.436–31.522	31.436	1.71E-04	5	*qCTS4-2*, L48 ^6)^, *qLTG-4*	LOC_Os04g52800
	*temperate japonica*	4	31.523–31.524	31.524	1.97E-04	3	*qCTS4-2*	LOC_Os04g52920
*qCTGERM5-1*	*tropical japonica*	5	21.435–21.485	21.484	3.56E-05	4	L51^5)^^,^^6)^, *qLTG5*-*2*^5)^, *qCTS5* ^5)^	LOC_Os05g36240
*qCTGERM6-1*	*JAPONICA*	6	6.014–6.143	6.014	8.21E-04	3	*qCTS6-1*^5)^, *COLD4*	LOC_Os06g11410
*qCTGERM6-2*	*ALL*	6	6.142–6.226	6.178	1.83E-04	6		LOC_Os06g11650
	*INDICA*	6	6.199–6.937	6.937	4.48E-04	5	*qCTS6-2*	
*qCTGERM7-1*	*tropical japonica*	7	10.464–10.647	10.525	1.02E-04	6	L72^5)^^,^ ^6)^, *qPSST-7* ^5)^, *qCTS7* ^5)^	
*qCTGERM7-2*	*tropical japonica*	7	13.211–13.359	13.359	4.01E-06	3		
*qCTGERM7-3*	*ALL*	7	17.133–17.353	17.343	7.08E-05	6	*qCTS7(2)*	
*qCTGERM7-4*	*ALL*	7	19.794–20.262	20.134	4.69E-05	15	*qCTS7(2)*, *qCTS7-2*	LOC_Os07g33690
	*JAPONICA*	7	19.587–20.171	20.000	8.39E-05	19	*qCTS7(2)*	LOC_Os07g33480, LOC_Os07g33600 LOC_Os07g33670
*qCTGERM7-5*	*tropical japonica*	7	27.860–27.947	27.947	4.01E-06	4	L79 ^6)^	
*qCTGERM8-1*	*ALL*	8	10.438–10.903	10.000	5.69E-05	7	*qCTF8*, *qCTS8-3*	LOC_Os08g17080
*qCTGERM8-2*	*JAPONICA*	8	12.270–12.345	12.270	8.85E-05	5		LOC_Os08g20420
*qCTGERM9-1*	*INDICA*	9	10.760–10.911	10.806	4.69E-05	7		LOC_Os09g17670
*qCTGERM10-1*	*ALL*	10	1.403–1.526	1.520	2.52E-04	3		
*qCTGERM10-2*	*ALL*	10	3.009–3.111	3.010	5.21E-04	*4*		
*qCTGERM10-3*	*tropical japonica*	10	10.165–10.335	10.257	2.32E-04	4	L102 ^6)^, *qLTG-10*	LOC_Os10g20390
*qCTGERM10-4*	*INDICA*	10	22.258–22.298	22.298	7.71E-06	3	*qCST10*, L107 ^6)^	
*qCTGERM11-1*	*temperate japonica*	11	1.6248–1.624	1.624	1.86E-04	4		
*qCTGERM11-2*	*ALL*	11	5.590–5.741	5.590	2.88E-04	5		
*qCTGERM11-3*	*JAPONICA*	11	16.230–16.248	16.248	2.41E-05	6		LOC_Os11g28270
*qCTGERM11-4*	*INDICA*	11	17.303–17.425	17.413	6.06E-05	7	*qCTS11-5*	LOC_Os11g29970
*qCTGERM11-5*	*JAPONICA*	11	19.676–19.770	19.718	7.78E-05	11		LOC_Os11g33330
	*temperate japonica*	11	19.675–19.757	19.718	4.65E-04	6	*qCTS11(2)-2*	
*qCTGERM12-1*	*JAPONICA*	12	24.896–24.904	24.904	3.53E-04	3		
*qCTGERM12-2*	*ALL*	12	26.314–26.426	26.430	2.55E-04	9	*qCTF12*	LOC_Os12g42540
	*INDICA*	12	26.353–26.489	26.366	5.20E-05	8	*qCTF12*	LOC_Os12g42420, LOC_Os12g42550
*qCTGERM12-3*	*INDICA*	12	27.195–27.281	27.219	4.20E-04	6	*qCTF12*	LOC_Os12g43840

^1)^ Additional information on the RDP1 accessions available on the GSOR website (https://www.ars.usda.gov/southeast-area/stuttgart-ar/dale-bumpers-national-rice-research-center/docs/rice-diversity-panel-1/)

^2)^ Most of these QTLs also are identified in GRAMENE [40].

^3)^ Additional information on the co-located QTLs is in S4 Table.

^4)^ Additional information on the co-located candidate genes is in S5 Table.

^5)^ SNP near to QTL.

^6)^ “L” is for Locus as reported by Lv et al. [13].

Several of the germination-related QTL regions identified in this study co-localized with previously reported QTLs, as summarized in Table 2 (S4 Table). The fact that these QTL regions were identified in multiple studies using different populations or collections of germplasm suggests the QTL should be explored further to validate their impact on cold tolerance at germination, and to clearly define the breeding value of the putative favorable allele(s), identified in this study. Three loci were significant across *ALL* and either *JAPONICA*, *temperate japonica* or *INDICA* and overlapped with the previously reported QTLs including *qCTGERM1-8* overlapping with *qCTS1-5*, *qCTGERM7-4* with *qCTS7(2)* and *qCTGERM12-2* with *qCTF12* (Table 2; S4 Table). Genomic positions of all germination index QTL are shown in Fig 3. Candidate genes proximal to each germination index QTL were determined by annotated gene function (S5 Table).

### Genome-wide association analysis at the reproductive stage

The GWA analysis was conducted to identify alleles associated with cold stress in the reproductive stage with MAF >0.05. A total of 31 QTL were identified including seven QTL associated with percent sterility, 14 with SWPlt, and 10 with SWPan (Table 3; Fig 3b). Some of these QTL co-localized with previously reported QTL associated with seed yield components (Table 3; S4 Table). Genomic positions of all reproductive stage cold tolerance QTL are shown alongside the germination index QTL in Fig 3 and candidate genes based on annotated gene function are listed in S5 Table.

**Table 3:** Summary of SNP positions for cold tolerance at the reproductive stage as identified by GWA mapping. Traits measured were percent sterility, seed weight per panicle and seed weight per plant. All SNPs above the threshold (p-value = 0.0001) are included. Reported QTLs and candidate genes associated with spikelet fertility and seed set which are co-located to these segments are identified.

QTL	Trait	Chromo-some	Position of segment (Mb)	SNP position (Mb)	p-value	No. significant SNPs	Co-located QTL^1)^^2)^	Co-located candidate gene ^3)^
*qSWTPNCT1-1*	Seed weight per panicle	1	3.235–3.451	3.452	4.29E-05	7	*Pdw1-1*^4)^	LOC_Os01g07310
*qSWTPNCT1-2*	Seed weight per panicle	1	22.238–23.466	22.287	3.33E-04	4		
*qSWTCT1-1*	Seed weight per plant	1	41.743–41.844	41.763	6.02E-06	27		LOC_Os01g72190
*qSWTCT1-2*	Seed weight per plant	1	41.847–41.999	41.895	1.02E-07	24	*qSW1-1*	LOC_Os01g72250
*qSWTCT1-3*	Seed weight per plant	1	42.033–42.102	42.099	1.52E-05	5		
*qSWTCT1-4*	Seed weight per plant	1	42.215–42.472	42.430	4.82E-05	9		
*qSWTPNCT1-3*	Seed weight per panicle	1	43.015–43.233	43.048	1.40E-04	35		LOC_Os01g74300
*qSWTPNCT2-1*	Seed weight per panicle	2	18.694–18.768	18.760	2.78E-04	8	*PNWT2*, *GWP2*^4)^	LOC_Os02g31280
*qFERCT2*	Percent sterility	2	19.320–19.427	19.425	3.16E-05	10	*nfg2*,*qLTG2*	LOC_Os02g32700
*qSWTCT4*	Seed weight per plant	2	25.010–25.047	25.017	2.20E-04	8		
*qSWTPNCT2-2*	Seed weight per panicle	2	28.617–29.067	29.198	7.64E-05	3	*yld2*.*1*, *qnob-5*	LOC_Os02g47744
*qSWTPNCT5-1*	Seed weight per panicle	5	27.018–27.195	27.195	1.14E-04	5	*qCTB5*^4)^	LOC_Os05g47470
*qSWTPNCT6-1*	Seed weight per panicle	6	5.927–6.193	6.194	1.52E-04	15	*qCTS6-1*^4)^	LOC_Os06g10470
*qFERCT6-1*	Percent sterility	6	8.313–8.339	8.330	5.75E-04	4		
*qFERCT6-2*	Percent sterility	6	13.419–13.557	13.467	1.36E-05	6		
*qFERCT6-3*	Percent sterility	6	14.520–15.627	14.774	6.08E-05	14		LOC_Os06g25250
*qFERCT6-4*	Percent sterility	6	16.654–16.765	16.659	1.61E-04	5	L63^5)^	
*qSWTCT6*	Seed weight per plant	6	24.582–24.749	24.711	2.96E-05	6	L66^5)^	LOC_Os06g41300
*qSWTPNCT6-2*	Seed weight per panicle	6	24.708–24.901	24.812	2.42E-04	8	*GWP6*	
*qSWTPNCT7-1*	Seed weight per panicle	7	1.700–1.764	1.761	5.15E-04	5	*qLTG7*	
*qFERCT7*	Percent sterility	7	14.316–14.471	14.316	3.44E-05	8	*PSS7*, *gp7*, L74^5)^	LOC_Os07g25060
*qSWTCT7*	Seed weight per plant	7	23.121–23.290	23.197	3.54E-05	7		LOC_Os07g38620
*qSWTCT8-1*	Seed weight per plant	8	0.628–0.886	0.688	2.39E-08	6		LOC_Os08g02094
*qSWTPNCT8-1*	Seed weight per panicle	8	2.914–2.942	2.942	6.44E-06	6		LOC_Os08g05510
*qSWTCT8-2*	Seed weight per plant	8	3.645–3.763	3.763	5.82E-05	7		LOC_Os08g06710
*qSWTCT8-3*	Seed weight per plant	8	6.209–6.270	6.248	3.20E-05	8		LOC_Os08g10608
*qSWTCT8-4*	Seed weight per plant	8	26.806–26.991	26.811	1.21E-04	6	L87^5)^	LOC_Os06g41300
*qSWTCT8-5*	Seed weight per plant	8	28.003–28.104	28.018	9.09E-05	16		LOC_Os08g44560
*qSWTCT9*	Seed weight per plant	9	16.540–16.678	16.540	1.27E-04	7		
*qFERCT12*	Percent sterility	12	7.234–7.347	7.243	4.17E-04	5	L125^5)^	
*qSWTCT12*	Seed weight per plant	12	26.116–26.129	26.123	6.82E-05	6	*gw12*.*1*	

^1)^ Most of these QTLs also are identified in GRAMENE [40].

^2)^ Additional information on the co-located QTLs is in S4 Table.

^3)^ Additional information on the co-located candidate genes is in S5 Table.

^4)^ SNP near to QTL.

^5)^ “L” is for Locus as reported by Lv et al. [13].

### Identification of candidate genes

The genome browser feature of Ricebase [42] was used to identify candidate genes based on the MSU7 gene annotation track within approximately 150kb of significant hits. Local pairwise linkage disequilibrium information (a custom Ricebase track) was used to guide selection of candidate genes. Gene ontology (GO) term enrichment analysis of the list of candidate genes indicated significant enrichment of response to stimuli (GO:0050896), response to biotic stimuli (GO:0009607), lipid metabolism (GO:0006629) and oxygen binding (GO:0019825) (e.g., cytochrome P450) genes (Table 4).

**Table 4:** Gene Ontology (GO) term enrichment analysis of the germination index candidate genes. The GO analysis describes the gene products in terms of their associated biological processes, cellular components and molecular functions independent of species.

GO term	Ontology	Description	Number in input list	Number annotated	p-value
GO:0006629	Biological process	lipid metabolic process	5	1376	0.0085
GO:0009607	Biological process	response to biotic stimuli	4	1404	0.0410
GO:0050896	Biological process	response to stimuli	11	6928	0.0440
GO:0019825	Molecular function	oxygen binding	4	390	0.0005

## Discussion

It can be challenging to map genetic loci controlling quantitative abiotic stress tolerance traits such as cold tolerance because they have a diffuse, polygenic architecture and are correlated with population structure. In this study, we applied high density genotyping along with GWA analysis corrected for population structure and kinship to elucidate the genetic control of cold tolerance in a collection of phenotypically and genetically diverse rice accessions. Twenty-eight regions associated with cold stress were identified across four subpopulations, both subspecies, and *ALL* RDP1 accessions.

Some of GWA mapped QTL associated with cold stress were located within or near to cold tolerance QTLs reported in bi-parental populations. The overlap provided confirmation of the GWA mapping results, and the GWA analysis allowed improved mapping resolution to the previously mapped loci. We found several peaks located within or near previously reported QTLs (Table 3; S4 Table) including 11 in *ALL* (*qCTB1* and *COLD3*, L21, *qCTS4-1*, *qCTS4-2*, *qCTS7*, *qCTS7-2*, *qCTF8*, *qCTS8-3*, *qCTS1-5*, *qCTS7-2*, and *qCTF12*), eight in *INDICA* (L21, L46, L107, *qCST10*, *qCTF12*, *qCTS11-5*, *qCTS6-2* and *qCTS4-3*), eight in *JAPONICA* [L8, L48, *qLTG-4*, *qCTS4-1*, *qCTS4-2*, *qCTS6-1*, *COLD4* and *qCTS7(2)*], four in *temperate japonica* [*qCTS4-1*, *qCTS4-2*, *qCTS1-5*, and *qCTS11(2)-2*] and 10 in *tropical japonica* (*qCTS5*, *qCTS10*, L51, *qLTG-5-2*, *qCTS5*, L72, *qPSST-7*, *qCTS7*, L79, L102, and *qLTG-10*). The QTLs, *qCTS4-1*, *qCTS4-2*, *COLD4*, *qCTB5* and *qCTS7(-2)* were identified in *JAPONICA* which is in agreement with our study [1, 6, 13, 32, 44–49]. Moreover, *qCTS4-1*and *qCTS4-2* are two major QTLs that were observed in *ALL*, *JAPONICA* and *temperate japonica;* another major QTL, *CTB7-2*, was detected in both *JAPONICA* and *ALL*; and finally a QTL, *qCTF12* was observed in both *INDICA* and *ALL*. There is a QTL region with a number of significant SNPs clustered between 4.178–4.180 Mb on chr. 1 observed in *ALL* and traced back to *JAPONICA* and the *temperate japonica* subpopulation (Table 2; Figs 2 and 3). Since no QTL was previously reported in this region, we suggest this region is a potential QTL region which should be further investigated.

At the reproductive stage, we detected several QTL located within or near previously reported QTLs associated with yield including five in percent sterility (*nfg2*, *PSS7*, *qLTG2*, L125, and *gp7*), four in SWPlt (*qSW1-1*, L66, L87, and *gw12*.*1*), and 11 in SWPan (*Pdw1-1*, *PNWT2*, *GWP2*, *yld2*.*1*, *qnob-5*, *qCTB5*, *qCTS6-1*, *GWP6*, *qple-4*, *qLTG7*, and *qCTSSR12-1* [1, 6, 13, 14, 31, 50–62]. The results showed that low temperature at the reproductive stage has a negative impact on the QTLs for yield associated traits. This finding is consistent with previous report by Andaya and Mackill [6] that low temperature occurring at the early reproductive stages causes much greater yield reduction than at the seedling stage.

The resolution of GWA analysis allows for the identification of candidate genes based on proximity to significant SNPs (S6 Table). Gene functional annotation revealed a number of plausible candidates. The gene annotations are based on the presence of protein domains and homology to genes with known function in rice or in other species. At both the molecular and cellular levels, several strategies, such as inducing biochemical and physiological changes, have been developed by plants in order to survive in cool and cold temperatures [63, 64]. Occurrence of low temperature causes variation in the expression of genes and alters the function of their product(s) to enhance tolerance to cold stress [65]. For example, the product of the candidate gene LOC_Os08g05510 belongs to the MYB transcription factor family. Previous studies showed that several MYB transcription factors such as AtMYB15 and AtMYB15 in *Arabidopsis*, as well as, MYBS3 in rice increase tolerance to cold stress [65,66]. The proteins produced by three candidate genes of LOC_Os11g28270, LOC_Os06g10470 and LOC_Os12g42540 belong to the zinc finger family. Proteins from the zinc finger family are overexpressed during cold stress in tobacco and rice [67,68]. The candidate genes LOC_Os09g17680, LOC_Os01g02750 and LOC_Os01g38850 belong to the kinase family which Shinozaki and Yamaguchi-Shinozaki [69] considered a potential signal of environmental stresses. The candidate gene LOC_Os07g46750 produces a protein that belongs to the elongation factor family which responds to cold stress in rice at the seedling stage as reported by Cui et al. [18]. Four candidate genes produce cytochrome P450 which Ito et al. [70] reported targets the stress inducible gene OsDREB1A whose protein promotes tolerance to cold and salt stresses in rice and *Arabidopsis* [71].

GO analysis revealed biological process and molecular function trends across all candidate genes. Lipid metabolic process was one of the enriched ontology terms. It is known that lipid metabolism has a role in the mechanism of cold tolerance in plants and acts by modulating the crystallization of lipids in plant membranes [72, 73]. Other significantly enriched biological process terms include response to stimuli and response to biotic stimuli. Although cold tolerance is an abiotic trait, there may be significant crosstalk with biotic stress response or presence of gene families that act in both forms of stress response [74, 75]. Oxygen binding was a significantly enriched molecular function ontology term. Oxygen binding proteins may provide protection from abiotic stress-induced oxidative damage or may be involved in the detection of reactive oxygen species to elicit a stress response [76].

Differences were found between the subspecies and subpopulations in their mean level of cold tolerance and in the genetic architecture of the cold tolerance within each group. The phenotypic study revealed that *JAPONICA* subspecies demonstrated more cold tolerance than the *INDICA* subspecies. We found only 12 *INDICA* but 87 *JAPONICA* accessions tolerant to cold stress. This finding is consistent with previous reports [13, 77, 78] that *INDICA* accessions are adapted to low latitude regions while *JAPONICA* accessions are adapted to low temperatures in high latitudes and higher elevations. These differences may be a result of adaptation to particular environments of subspecies progenitors prior to domestication or the result of human selection acting on the subspecies to optimize production in the regions were they are currently grown [79]. Furthermore, our results showed that the degree of tolerance in the studied subpopulations with *tropical japonica* being more tolerant than *temperate japonica* which in turn is more tolerant than *aus* and *indica*, is in agreement with the results of their attributed subspecies and with the seedling cold tolerance evaluation of RDP1 accessions [32]. In this particular rice collection, RDP1, the number of cold tolerant *tropical japonica* accessions was greater than *temperate japonica* accessions, but that order can be different in other rice collections. There was no statistically significant difference between *aus* and *indica*, although *aus* (0.46 + 0.03) was slightly more cold tolerant than *indica* (0.45 + 0.03). These results suggest that more alleles conferring cold tolerance at cold tolerance loci can be found in *JAPONICA* subspecies and its subpopulations than in *INDICA* subspecies and its subpopulations.

Given that there are differences in the genetic architecture of cold tolerance between subspecies and subpopulations (i.e., different genetic loci are involved), this presents an opportunity for enhancing cold tolerance through breeding. SNPs that are linked to cold tolerance alleles could serve as tools for marker assisted selection (MAS) in rice breeding programs. Pyramiding cold tolerance alleles at multiple genes would result in new rice varieties with enhanced cold tolerance. Transgressive variation for cold tolerance would be likely in crosses between subspecies through additive effects at multiple loci [80]. The results of this study also may guide the selection of optimum parental lines to use in plant breeding efforts focused on enhancing the cold tolerance of new rice varieties.

### Conclusion

In light of new technology, powerful statistical software and high density genotyping, it is possible to analyze complex quantitative traits with GWA mapping. In this study, we showed how GWA mapping can serve as a powerful tool by providing insight into the genetic architecture of cold tolerance in a diverse collection of rice genotypes by detecting QTLs and candidate genes associated with cold tolerance at germination and booting. Moreover, we demonstrated that the GWA mapping can complement and validate the previous cold tolerance QTL mapping studies conducted in bi-parental populations.

## Supporting information

S1 Table List of all significant SNPs identified by GWA analysis. Complete list of the all the significant SNPs identified for germination index by subpopulation group (*ALL* RDP1, *INDICA*, *JAPONICA*, *temperate japonica* and *tropical japonica*) and for the three reproductive traits evaluated, percent sterility, mean of seed weight per plant (SWPlt) and mean seed weight per panicle (SWPan).
(XLSX)

S2 Table List of the RDP1 accessions which were cold tolerant at germination. The 83 accessions listed had a germination index at least one standard error above the cold tolerant control, Quilla66304. (The three controls replicated across this experiment are listed at the end of the table.).
(PDF)

S3 Table List of the 206 RDP1 accessions identified evaluated for cold tolerance at the reproductive stage based on percent sterility, seed weight per plant or seed weight per panicle. The accessions are grouped as those a) greater than or equal to the most cold tolerant control, Mustakilik, for at least one measure of cold tolerance, b) less than Mustakilik for all cold tolerant traits measured, c) accessions which did not produce panicles and d) controls. Days to heading and the germination index are included as a reference.
(PDF)

S4 Table Summary of QTLs identified by GWA mapping in this study which co-localized with previously reported QTLs at the germination and reproductive stages. The previously reported QTLs were identified in both bi-parental populations and by GWA mapping in or near the QTL regions identified in this study.
(PDF)

S5 Table Summary of candidate genes co-located with SNPs identified in this study as associated with cold tolerance at the germination and reproductive stages. These candidate genes were verified as part of cold tolerance studies in other plant species.
(PDF)

S6 Table Overlap between candidate genes and significant SNPs. Regions where position of significant SNPs (Table 2) overlapped with the candidate genes (S5 Table) based on the germination index across the population groups.
(PDF)

S1 Fig Frequency distribution of the adjusted cold germination ratio for the RDP1 *O*. *sativa* accessions. Distribution of the “Germination Index” calculated as percentage of seeds with coleoptiles >5 mm in cold treatment to percentage of seeds with coleoptiles >5 mm in warm treatment, across *ALL* RDP1 accessions is the sum of the gray and hash bars. The hash bars indicate the frequency distribution for the subset of A) *INDICA* (*indica*, *aus*, admix-*INDICA*) accessions and B) *JAPONICA* (*tropical japonica*, *temperate japonica*, admix-*JAPONICA*) accessions. Quantile box-and-whisker plots show the distribution observed values and the mean for the *INDICA* and *JAPONICA* accessions. The cold tolerant check, Quilla66304 is shown. (Table 1 lists additional statistics.).
(PDF)

S2 Fig Distribution of the *JAPONICA* RDP1 accessions for three reproductive traits as a measure of cold tolerance at booting. The distribution of the mean percent sterility, [classified as category 1 (0 to 5% sterility), 2 (6 to 12%), 3 (13 to 18%), 4 (19 to 40%), 5 (41 to 60%), 6 (61 to 90%) and 7 (91 to 100%)], mean seed weight per panicle and mean seed weight per plant as a measure of cold tolerance at booting (panicle emergence) in rice. Quantile box-and-whisker plots show the distribution of observed values and the mean for each trait. The value for cold tolerant check, Mustakilik, is shown. (Table 1 lists additional statistics.).
(PDF)

S3 Fig Quantile-Quantile plots for four cold tolerance traits measured at germination and at the reproductive stages. The *y*-axis is the observed number and magnitude of observed *p*-value (-10 log) displays associations between SNPs and cold tolerance and the *x*-axis is the expected *p-*value (-10 log) under the null hypothesis (there is no association between SNPs and cold tolerance). The λ (lambda) is the genomic inflation factor.
(PDF)
