# Supplementary material for: Genetic architecture of cold tolerance in rice (Oryza sativa) determined through high resolution genome-wide analysis
Source: PLoS One. 2017 Mar 10;12(3):e0172133. doi: 10.1371/journal.pone.0172133 (PMC5345765; doi:10.1371/journal.pone.0172133)
Supplement: S2 Table — The 83 accessions listed had a germination index at least one standard error above the cold tolerant control, Quilla66304. (The three controls replicated across this experiment are listed at the end of the table.). (PDF) [file pone.0172133.s002.pdf]

S2 Table. List of the RDP1 accessions which were cold tolerant at germination. The 83 accessions listed had a germination index at least one standard error above the cold tolerant control, Quilla66304. (The three controls replicated across this experiment are listed at the end of the table.)

Rank	Accession [1)]	Germination index [2), 3)]	Subpopulation group [4)]	Subspecies	Original providing country [4)]	Region/Originating country
1	NPE 835	2.67	*temperate japonica*	*JAPONICA*	Pakistan	South Asia
2	Haginomae Mochi	1.71	*temperate japonica*	*JAPONICA*	Japan	East Asia
3	Llanero 501	1.17	*tropical japonica*	*JAPONICA*	Zaire	South America
4	Li-Jiang-Xin-Tuan-Hei-Gu	1.10	*temperate japonica*	*JAPONICA*	China	East Asia
5	Romeo	1.08	*temperate japonica*	*JAPONICA*	Italy	West Europe
6	Triomphe Du Maroc	1.06	*temperate japonica*	*JAPONICA*	Morocco	Africa
7	Edith	1.05	*tropical japonica*	*JAPONICA*	United States	North America
8	Karabaschak	1.05	*temperate japonica*	*JAPONICA*	Bulgaria	East Europe
9	Early	1.03	*admixed-japonica*	*JAPONICA*	China	North America
10	LaGrue	1.03	*tropical japonica*	*JAPONICA*	United States	North America
11	M-202	1.03	*temperate japonica*	*JAPONICA*	United States	North America
12	Baber	1.02	*temperate japonica*	*JAPONICA*	India	South Asia
13	318	1.02	*tropical japonica*	*JAPONICA*	India	West Asia
14	Kinastano	1.02	*tropical japonica*	*JAPONICA*	Philippines	Southeast Asia
15	Jambu	1.02	*tropical japonica*	*JAPONICA*	Indonesia	Southeast Asia
16	TKM6	1.01	*indica*	*INDICA*	India	South Asia
17	Niquen	1.01	*temperate japonica*	*JAPONICA*	Chile	South America
18	Spring	1.01	*tropical japonica*	*JAPONICA*	United States	North America
19	Padi Pagalong	1.00	*tropical japonica*	*JAPONICA*	Malaysia	Southeast Asia
20	Kibi	1.00	*temperate japonica*	*JAPONICA*	Japan	East Asia
21	NSF-TV 107	1.00	*tropical japonica*	*JAPONICA*	Unknown	Unknown
22	Priano Guaira	1.00	*tropical japonica*	*JAPONICA*	Brazil	South America
23	Manzano	1.00	*tropical japonica*	*JAPONICA*	Zaire	Africa
24	Bengal	1.00	*admixed-japonica*	*JAPONICA*	United States	North America
25	Saber	1.00	*tropical japonica*	*JAPONICA*	United States	North America
26	Chinese	0.99	*temperate japonica*	*JAPONICA*	China	East Asia
27	Luk Takhar	0.99	*temperate japonica*	*JAPONICA*	Afghanistan	South Asia
28	Guineandao	0.99	*admixed-japonica*	*JAPONICA*	Guinea	Africa
29	Pate Blanc Mn 1	0.98	*tropical japonica*	*JAPONICA*	Cote D'Ivoire	Africa

30	325	0.98	*tropical japonica*	*JAPONICA*	Taiwan	East Asia
31	Carolina Gold 12034	0.98	*tropical japonica*	*JAPONICA*	United States	North America
32	Taipei 309	0.98	*temperate japonica*	*JAPONICA*	Taiwan	East Asia
33	Wells	0.98	*tropical japonica*	*JAPONICA*	United States	North America
34	British Honduras Creole	0.97	*tropical japonica*	*JAPONICA*	Belize	Central America
35	Hu Lo Tao	0.97	*temperate japonica*	*JAPONICA*	China	East Asia
36	Sab Ini	0.97	*temperate japonica*	*JAPONICA*	Egypt	Africa
37	Sinaguing	0.97	*tropical japonica*	*JAPONICA*	Philippines	Southeast Asia
38	Vary Vato 462	0.97	*admixed-indica*	*INDICA*	Madagascar	Africa
39	PR 304	0.96	*tropical japonica*	*JAPONICA*	Puerto Rico	Caribbean
40	Ai-Chiao-Hong	0.96	*indica*	*INDICA*	China	East Asia
41	Saturn	0.96	*tropical japonica*	*JAPONICA*	United States	North America
42	Melanotrix	0.96	*temperate japonica*	*JAPONICA*	Tajikistan	Central Asia
43	IAC 25	0.96	*tropical japonica*	*JAPONICA*	Brazil	South America
44	Ta Mao Tsao	0.96	*temperate japonica*	*JAPONICA*	China	East Asia
45	Guatemala 1021	0.96	*tropical japonica*	*JAPONICA*	Guatemala	Central America
46	NSF-TV 93	0.95	*aromatic*	*JAPONICA*	Unknown	Unknown
47	Doble Carolina Rinaldo Barsani	0.95	*admixed-japonica*	*JAPONICA*	Uruguay	South America
48	Mojito Colorado	0.95	*tropical japonica*	*JAPONICA*	Bolivia	South America
49	Okshitmayin	0.95	*admixed-japonica*	*JAPONICA*	Myanmar	Southeast Asia
50	Upland	0.95	*tropical japonica*	*JAPONICA*	Ponape Island, Micronesia	Oceania
51	Carolina Gold Sel	0.95	*tropical japonica*	*JAPONICA*	United States	North America
52	Yabani Montakhab 7	0.95	*temperate japonica*	*JAPONICA*	Egypt	Africa
53	JC149	0.95	*indica*	*INDICA*	India	South Asia
54	Keriting Tingii	0.95	*admixed-japonica*	*JAPONICA*	Indonesia	Southeast Asia
55	Nova	0.95	*admixed-japonica*	*JAPONICA*	United States	North America
56	Rikuto Norin 21	0.95	*admixed-japonica*	*JAPONICA*	Japan	East Asia
57	OS 6 (WC 10296)	0.94	*tropical japonica*	*JAPONICA*	Zaire	Africa
58	Baghlani Nangarhar	0.94	*temperate japonica*	*JAPONICA*	Afghanistan	South Asia
59	Sanbyang-Daeme	0.94	*admixed-japonica*	*JAPONICA*	Republic of Korea	East Asia
60	IRAT 13	0.94	*tropical japonica*	*JAPONICA*	Cote D'Ivoire	Africa
61	KU115	0.94	*tropical japonica*	*JAPONICA*	Thailand	Southeast Asia

62	Italica Carolina	0.94	*temperate japonica*	*JAPONICA*	Poland	East Europe
63	Sintane Diofor	0.93	*indica*	*INDICA*	Burkina Faso	Africa
64	Taichung Native 1	0.93	*indica*	*INDICA*	Taiwan	East Asia
65	Nipponbare	0.93	*temperate japonica*	*JAPONICA*	Japan	East Asia
66	Azucena	0.93	*tropical japonica*	*JAPONICA*	Philippines	Southeast Asia
67	WC 4443	0.93	*tropical japonica*	*JAPONICA*	Bolivia	South America
68	WC 4419	0.93	*tropical japonica*	*JAPONICA*	Honduras	Central America
69	Sundensis	0.93	*indica*	*INDICA*	Kazakhstan	Central Asia
70	Shirogane	0.93	*temperate japonica*	*JAPONICA*	Japan	East Asia
71	CI 11026	0.92	*admixed*	*admixed*	United States	North America
72	Taducan	0.92	*indica*	*INDICA*	Philippines	Southeast Asia
73	Jefferson	0.92	*tropical japonica*	*JAPONICA*	United States	North America
74	WAB 56-104	0.92	*tropical japonica*	*JAPONICA*	Cote D'Ivoire	Africa
75	Khao Pahk Maw	0.92	*aus*	*INDICA*	Thailand	Southeast Asia
76	Bahia	0.91	*temperate japonica*	*JAPONICA*	Spain	West Europe
77	Tropical Rice	0.91	*temperate japonica*	*JAPONICA*	Ecuador	South America
78	Blue Rose	0.91	*admixed-japonica*	*JAPONICA*	United States	North America
79	Boa Vista	0.91	*tropical japonica*	*JAPONICA*	El Salvador	Central America
80	Pai Hok Glutinous	0.91	*indica*	*INDICA*	Hong Kong	East Asia
81	JC 117	0.91	*indica*	*INDICA*	India	South Asia
82	Sml Kapuri	0.91	*temperate japonica*	*JAPONICA*	Suriname	South America
83	Kaw Luyoeng	0.91	*temperate japonica*	*JAPONICA*	Thailand	Southeast Asia
**Controls**						
	Quilla 66304	0.89	High tolerance			
	Lemont	0.45	Intermediate tolerance			
	Zhe733	0.29	Low tolerance			

[1] Additional information on the RDP1 accessions available on the GSOR website (https://www.ars.usda.gov/southeast-area/stuttgart-ar/dale-bumpers-national-rice-research-center/docs/rice-diversity-panel-1/)

[2] The germination index is calculated as the ratio of the mean percentage of seeds with coleoptiles >5 mm under cold treatment to the mean percentage of seeds with coleoptiles >5 mm under warm treatment.

[3] For *ALL* RDP1 accessions the mean germination index was 0.63 and the Standard Error was 0.02.

[4] The subpopulation group and original providing country are listed as previously reported (4, 33).
