# Supplementary material for: Genetic architecture of cold tolerance in rice (Oryza sativa) determined through high resolution genome-wide analysis
Source: PLoS One. 2017 Mar 10;12(3):e0172133. doi: 10.1371/journal.pone.0172133 (PMC5345765; doi:10.1371/journal.pone.0172133)
Supplement: S3 Table — The accessions are grouped as those a) greater than or equal to the most cold tolerant control, Mustakilik, for at least one measure of cold tolerance, b) less than Mustakilik for all cold tolerant traits measured, c) accessions which did not produce panicles and d) controls. Days to heading and the germination index are included as a reference. (PDF) [file pone.0172133.s003.pdf]

S3 Table. List of the 206 RDP1 accessions identified evaluated for cold tolerance at the reproductive stage based on percent sterility, seed weight per plant or seed weight per panicle. The accessions are grouped as those a) greater than or equal to the most cold tolerant control, Mustakilik, for at least one measure of cold tolerance, b) less than Mustakilik for all cold tolerant traits measured, c) accessions which did not produce panicles and d) controls. Days to heading and the germination index are included as a reference.

Accession [1)]	Germination index	Heading date	Percent sterility [2)]	Seed weight (g) per plant	Seed weight (g) per panicle
**Accessions which were more cold tolerant for at least one trait measured [3)]**					
27	0.76	81	***2.5***	6.05	1.13
Agostano	0.71	75	***3.0***	***11.95***	1.37
Agusita	0.54	60	***2.5***	***15.10***	***1.41***
Aichi Asahi	0.87	136	***2.5***	2.95	0.39
ASD 1	0.70	86	***3.0***	***15.00***	1.09
Azerbaidjanica	0.50	56	***3.0***	7.50	0.83
Azucena_312001	0.87	136	***2.5***	4.15	0.64
Baber	***1.02***	79	***2.0***	11.05	1.11
Bahia	***0.91***	130	***2.0***	3.20	0.64
Baldo	0.61	75	4.5	6.80	***1.54***
Bellardone	0.72	73	3.5	10.25	***1.59***
Bergreis	0.71	136	***2.0***	6.00	0.92
Biser 1	0.73	77	***1.5***	***14.80***	***1.96***
Blue Rose Supreme	0.82	134	***2.5***	5.70	1.07
Boa Vista	***0.91***	140	***2.5***	1.40	0.40
Bul Zo	0.02	135	***3.0***	4.80	0.83
Bulgare	0.87	48	***2.0***	6.00	0.70
Chodongji	0.74	126	***2.5***	7.40	0.99
CS-M3	0.20	133	***2.5***	6.25	0.67
Early Wataribune	0.87	113	***3.0***	5.95	0.91
Erythroceros Hokkaido	0.75	48	***2.5***	6.10	0.92
Gambiaka Sebela	0.48	134	***2.5***	1.85	0.57
Hiderisirazu	0.86	82	***3.0***	7.50	1.36
IAC 25	***0.96***	73	***2.0***	6.80	1.13
Iguape Cateto	0.61	134	***2.0***	5.25	1.32
IITA 135	0.71	128	***3.0***	2.25	1.13
Italica Carolina	***0.94***	53	***2.0***	9.70	1.01
Kamenoo	0.83	78	***3.0***	9.70	0.94
Karabaschak	***1.05***	77	***3.0***	***17.75***	***1.57***
Kihogo	0.67	130	***2.0***	4.70	1.03
Kiuki No. 46	0.88	128	***2.5***	1.95	0.31

Kon Suito	0.77	43	***2.5***	6.95	0.60
Koshihikari	0.67	141	***3.0***	0.55	0.22
Leuang Hawn	0.43	45	***2.5***	3.45	0.67
Ligerito	0.78	76	***3.0***	5.75	1.04
Li-Jiang-Xin-Tuan-Hei-Gu	***1.10***	103	***3.0***	10.85	1.26
Lusitano	0.69	56	4.0	6.30	***1.41***
M. Blatec	0.61	82	***3.0***	***20.45***	***2.56***
M-202_301370	0.75	124	***2.5***	1.95	0.49
M-202_312011	***1.03***	119	***3.0***	5.90	0.87
Mansaku	0.45	79	***2.5***	6.75	1.13
Manzano	***1.00***	110	***3.0***	3.00	0.41
Melanotrix	***0.96***	71	***3.0***	11.45	1.15
Nipponbare_301164	***0.93***	134	***3.0***	1.40	0.16
Norin 20	0.53	46	***3.0***	5.45	1.01
Nortai	0.61	135	***2.5***	2.35	0.57
Nucleoryza	0.90	55	***2.5***	11.25	0.77
Oro	0.36	76	***2.5***	***14.50***	***1.71***
OS 6 (WC 10296)	***0.94***	121	***2.5***	5.30	0.76
Padi Pagalong	***1.00***	103	***2.5***	3.80	0.80
Panda	0.86	110	***1.5***	***13.95***	***1.46***
PI 298967-1	0.41	141	***2.0***	2.00	0.46
Priano Guaira	***1.00***	72	***2.0***	6.00	0.90
Rikuto Kemochi	0.48	99	3.5	8.60	***1.60***
Rikuto Norin 21	***0.95***	80	***1.5***	***12.25***	***1.70***
Romanica	0.68	49	***2.0***	11.60	1.16
Sab Ini	***0.97***	72	***3.0***	***12.40***	1.15
Sanbyang-Daeme	***0.94***	105	***2.5***	4.75	0.86
Shinriki	0.36	135	***2.0***	3.15	0.48
Sitpwa	0.54	105	***3.0***	5.50	0.92
Sml Kapuri	***0.91***	110	***3.0***	5.50	0.93
Sri Malaysia Dua	0.23	76	***3.0***	10.10	0.84
Stegaru 65	0.46	65	***3.0***	***14.25***	1.16
Ta Hung Ku	0.64	88	4.0	***12.35***	0.94
Tokyo Shino Mochi	0.78	99	***3.0***	6.70	1.03
Tropical Rice	***0.91***	56	4.0	***11.85***	***1.41***
Vavilovi	0.83	53	***3.0***	***13.75***	1.05
Vialone	0.80	90	5.0	8.40	***1.40***
Victoria F.A.	0.73	78	4.0	***11.95***	***1.45***
WAB 56-104	***0.92***	74	***3.0***	7.80	***1.38***
WIR 3039	0.84	71	***3.0***	8.55	0.73
WIR 3764	0.46	50	***3.0***	9.85	0.53

YRL-1	0.84	110	3.5	10.50	***<u>1.39</u>***
**<u>Accessions which did not show cold tolerance at the reproductive stage</u>**					
318	***<u>1.02</u>***	130	4.0	1.65	0.57
325	***<u>0.98</u>***	128	3.5	4.30	0.96
519	0.36	90	7.0	0.10	0.01
56-122-23	0.38	110	4.5	3.65	0.33
68-2	0.68	95	4.5	4.55	0.58
Amposta	0.21	76	5.0	2.60	0.27
Azucena_301165	***<u>0.93</u>***	131	4.5	1.10	0.24
B6616A4-22-Bk-5-4	0.77	85	6.0	0.85	0.14
Baghlani Nangarhar	***<u>0.94</u>***	123	4.0	1.40	0.14
Bengal	***<u>1.00</u>***	127	4.5	5.25	0.76
Benllok	0.63	108	3.5	8.65	0.93
Beonjo	0.73	125	4.0	4.95	0.41
Blue Rose	***<u>0.91</u>***	135	4.0	5.55	0.94
Bombilla	0.53	116	6.5	0.45	0.06
Bombon	0.59	122	4.0	6.95	1.20
Breviaristata	0.86	86.5	4.5	6.65	1.11
British Honduras Creole	***<u>0.97</u>***	81	5.0	4.20	0.50
C57-5043	0.70	86	4.5	3.60	0.51
Carolina Gold 12033	0.85	128	5.5	1.80	0.50
Carolina Gold 12034	***<u>0.98</u>***	130	5.0	3.40	0.55
Carolina Gold Sel	***<u>0.95</u>***	129	5.0	2.00	0.55
Caucasica	0.69	101	3.5	6.35	1.33
Cenit	0.79	128	3.5	4.80	0.88
Chibica	0.90	106	4.0	10.65	0.85
Cocodrie	0.30	88.5	5.0	0.95	0.16
Creole	0.84	129	5.0	2.15	0.39
Cuba 65	0.85	98	5.0	2.50	0.32
Cybonnet	0.01	87	7.0	0.10	0.02
Cypress	0.43	106	6.5	0.55	0.06
Dam	0.33	133	3.5	3.95	0.56
Darmali	0.68	129	4.0	5.25	0.83
Della	0.49	130	4.5	1.90	0.30
Deokjeokjodo	0.69	116	4.0	2.60	0.43
Desvauxii	0.88	81	4.5	4.90	0.58
Doble Carolina Rinaldo Barsani	***<u>0.95</u>***	93	4.5	7.15	0.97
Dosel	0.54	120	4.0	3.75	0.70
Dourado Agulha	0.21	135	4.5	3.20	0.57

Early	***<u>1.03</u>***	62	4.5	7.30	0.77
Edith	***<u>1.05</u>***	121	5.5	3.55	0.47
Edomen Scented	0.58	137	3.5	2.00	0.24
Estrela	0.79	89	5.0	2.45	0.30
Fossa Av	0.59	129	5.5	1.45	0.23
Gogo Lempuk	0.87	129	5.5	0.40	0.08
Gotak Gatik	0.73	89	6.0	0.10	0.03
Guatemala 1021	***<u>0.96</u>***	119	5.0	3.30	0.47
Guineandao	***<u>0.99</u>***	128	3.5	4.35	0.61
Gyehwa 3	0.42	121	5.5	0.45	0.14
H256-76-1-1-1	0.87	105	5.0	1.25	0.31
Habiganj Boro 6	0.34	90	5.0	5.55	0.59
Haginomae Mochi	***<u>1.71</u>***	120	3.5	3.15	0.53
Hatsunishiki	0.82	101	5.5	2.40	0.23
Heukgyeong	0.77	104	3.5	9.05	0.87
Honduras	0.85	134	4.0	3.95	0.73
IRAT 44	0.80	91	4.0	1.50	0.38
Jambu	***<u>1.02</u>***	126	7.0	0.15	0.10
Jefferson	***<u>0.92</u>***	86	6.5	1.80	0.53
Jouiku 393G	0.27	83	4.5	5.60	0.76
Kaniranga	0.89	78	4.5	3.25	0.66
Katy	0.31	116	6.5	0.25	0.11
Kaw Luyoeng	***<u>0.91</u>***	135	4.5	2.70	0.59
Kaybonnet	0.89	89	7.0	0.10	0.02
Keriting Tingii	***<u>0.95</u>***	138	4.0	2.05	0.47
Kinastano	***<u>1.02</u>***	133	5.0	2.10	0.70
L-202	0.73	82	4.5	3.80	0.68
LAC 23	0.02	134	4.0	2.65	0.75
Lacrosse	0.83	135	4.5	4.75	0.76
Lady Wright Seln	0.78	105	4.5	5.40	0.77
Lomello	0.82	87	5.0	3.20	0.62
Maratelli	0.57	76	4.5	6.70	1.34
Mojito Colorado	***<u>0.95</u>***	132	4.0	3.65	0.52
Nipponbare_312005	0.84	121	6.5	0.50	0.06
Niquen	***<u>1.01</u>***	47	3.5	5.25	0.76
Nova	***<u>0.95</u>***	132	4.5	3.85	0.59
NSF-TV 107	***<u>1.00</u>***	75	4.0	5.15	0.86
NSF-TV 116	0.51	126	4.5	3.40	0.58
NSF-TV 199	0.72	79	7.0	0.10	0.02
NSF-TV 89	0.71	121	5.0	0.80	0.20
NSF-TV 95	0.84	129	6.5	0.95	0.21

Okshitmayin	***0.95***	118	5.0	1.55	0.15
OS6	0.61	121	5.0	1.35	0.26
Osogovka	0.31	101	4.5	5.25	1.02
Ostiglia	0.81	124	4.0	2.90	0.41
Palmyra	0.84	125	3.5	6.15	0.95
Pate Blanc Mn 1	***0.98***	139	4.0	0.95	0.27
Patna	0.33	107	5.0	2.60	0.54
Pato De Gallinazo	0.46	84	4.5	5.25	0.56
Pecos	0.67	122	5.0	2.90	0.39
PR 304	***0.96***	119	4.5	3.65	0.56
Pratao	0.70	133	4.0	2.25	0.78
R 101	0.80	141	5.0	1.60	0.34
Razza 77	0.57	105	3.5	6.95	1.08
Rinaldo Bersani	0.89	105	4.5	6.60	0.69
Romeo	***1.08***	94	5.0	5.70	1.27
Rosemont	0.67	90	5.0	1.30	0.31
Saber	***1.00***	88	3.5	2.05	0.44
Saku	0.38	107	4.5	2.90	0.56
Saturn	***0.96***	126	5.0	1.85	0.40
Shirogane	***0.93***	78	6.5	2.40	0.13
Shoemed	0.37	132	3.5	2.60	0.52
Sung Liao 2	0.37	63	6.0	1.65	0.21
Ta Mao Tsao	***0.96***	105	4.5	4.65	0.49
Tia Bura	0.85	122	5.0	2.55	0.36
Toploea 70/76	0.49	57	5.0	0.60	0.08
Trembese	0.25	134	7.0	0.30	0.09
Upland	***0.95***	128	4.0	3.10	0.74
Uzbekskij 2	0.23	78	4.5	5.60	1.00
Varyla	0.79	86	3.5	4.00	0.68
WAB 501-11-5-1	0.54	96	5.5	2.35	0.30
WAB 502-13-4-1	0.79	88	4.0	6.10	0.97
Wanica	0.90	126	5.5	3.40	0.50
WC 3397	0.90	71	7.0	0.10	0.02
WC 521	0.42	114	4.5	1.95	0.44
WC 6	0.51	112	4.5	4.40	0.55
**Accessions which did not head during treatment period** [4)]					
ARC 10086	0.85	_	_	_	_
Arias	0.85	_	_	_	_
Asse Y Pung	0.82	_	_	_	_
C1-6-5-3	0.80	_	_	_	_

Caawa/Fortuna 6-103-15	0.01	_	_	_	_
Canella De Ferro	0.90	_	_	_	_
Chinese	***<u>0.99</u>***	_	_	_	_
CI 11011	0.56	_	_	_	_
Delrex	0.90	_	_	_	_
Fortuna	0.54	_	_	_	_
Geumobyeo	0.18	_	_	_	_
Hu Lo Tao	***<u>0.97</u>***	_	_	_	_
IRAT 13	***<u>0.94</u>***	_	_	_	_
IRAT 177	0.84	_	_	_	_
Kibi	***<u>1.00</u>***	_	_	_	_
KU115	***<u>0.94</u>***	_	_	_	_
LaGrue	***<u>1.03</u>***	_	_	_	_
Leah	0.76	_	_	_	_
Lemont	0.69	_	_	_	_
Llanero 501	***<u>1.17</u>***	_	_	_	_
Luk Takhar	***<u>0.99</u>***	_	_	_	_
MCR010277	0.00	_	_	_	_
Moroberekan_301100	0.82	_	_	_	_
Moroberekan_312004	0.84	_	_	_	_
NSF-TV 27	0.46	_	_	_	_
Padi Kasalle	0.72	_	_	_	_
RT 1031-69	0.85	_	_	_	_
S4542A3-49B-2B12	0.73	_	_	_	_
Shangyu 394	0.53	_	_	_	_
Sinaguing	***<u>0.97</u>***	_	_	_	_
Sinampaga Selection	0.90	_	_	_	_
Suweon§§	0.75	_	_	_	_
Tainan Iku 487	0.60	_	_	_	_
Tainan-Iku No. 512	0.46	_	_	_	_
Tainung 67	0.33	_	_	_	_
Taipei 309	***<u>0.98</u>***	_	_	_	_
Tondok	0.73	_	_	_	_
Triomphe Du Maroc	***<u>1.06</u>***	_	_	_	_
WC 4419	***<u>0.93</u>***	_	_	_	_
WC 4443	***<u>0.93</u>***	_	_	_	_
Yabani Montakhab 7	***<u>0.95</u>***	_	_	_	_
**<u>Controls</u>**	_				
Mustakilik	_	112	3.5	11.33	1.34
M-202	_	125	3.5	7.50	0.69

M-206	_	89	3.5	6.03	0.67
M-205	_	113	4.0	3.98	0.43
L-206	_	86	4.3	2.50	0.31
Calmochi 101	_	90	4.8	2.20	0.21
Calmati 201	_	94	6.3	2.60	0.22
Jinbu	_	73	7.0	0.50	0.06

[1] Accessions underscored were cold tolerant for the three reproductive traits and bold, italics indicates cold tolerant at germination too.

[2] Percent sterility was rated with the following categories: category 1 (0 to 5% sterility), 2 (6 to 12%), 3 (13 to 18%), 4 (19 to 40%), 5 (41 to 60%), 6 (61 to 90%) and 7 (91 to 100%).

[3] All traits in bold, italics and underscored were better than the most tolerant check the SE (Table 1). SE values: percent sterility (0.1), seed weight per plant (0.28) and seed weight per panicle (0.03)

[4] Dash indicates no data was collected for the given trait.
