# Supplementary material for: Genetic architecture of cold tolerance in rice (Oryza sativa) determined through high resolution genome-wide analysis
Source: PLoS One. 2017 Mar 10;12(3):e0172133. doi: 10.1371/journal.pone.0172133 (PMC5345765; doi:10.1371/journal.pone.0172133)
Supplement: S4 Table — The previously reported QTLs were identified in both bi-parental populations and by GWA mapping in or near the QTL regions identified in this study. (PDF) [file pone.0172133.s004.pdf]

S4 Table. Summary of QTLs identified by GWA mapping in this study which co-localized with previously reported QTLs at the germination and reproductive stages. The previously reported QTLs were identified in both bi-parental populations and by GWA mapping in or near the QTL regions identified in this study.

QTL	Population group /Trait [1)]	QTL	Chr.	QTL region (bp)	Marker position (bp)	Description [2)]	Reference[3),4),5)]
**Germination**							
qCTGERM1-3	*ALL*	*qCTB1* [6)]	1	5,558,576-7,445,919	8,190,528	cold tolerance	6
	ALL	*COLD3*	1	7,280,970-9,390,430	8,190,528	cold tolerance	47
qCTGERM1-6	*JAPONICA*	L8 [7)]	1	22,610,678	22,610,678	ELC2	13
qCTGERM1-8	*ALL*	*qCTS1-5*	1	41,860,220	41,860,220	cold tolerance	32
	temperate japonica	*qCTS1-5*	1	41,860,220	41,860,220	cold tolerance	32
qCTGERM2-2	*ALL*	L21 [7)]	2	6,695,685	6,695,685	ELSR2	13
	INDICA	L21 [7)]	2	6,695,685	6,695,685	ELSR2	13
qCTGERM4-2	*ALL*	*qCTS4-1*	4	688,353-6,574,518	1,116,687	cold tolerance	1
	JAPONICA	*qCTS4-1*	4	688,353-6,574,518	866,416	cold tolerance	1, 55, 82
	temperate japonica	*qCTS4-1*	4	688,353-6,574,518	894,359	cold tolerance	1, 55, 82
qCTGERM4-3	*INDICA*	L46 [7)]	4	27,105,852	27,105,852	RLC	1, 13
	INDICA	*qCTS4-3*	4	26,857,374-29,061,127	27,214,088	cold tolerance	1
qCTGERM4-4	*ALL*	*qCTS4-2*	4	30,772,388-32,650,528	31,159,129	cold tolerance	1, 55
	JAPONICA	*qCTS4-2*	4	30,772,388-32,650,528	31,436,463	cold tolerance	82
	temperate japonica	*qCTS4-2*	4	30,772,388-32,650,528	31,524,171	cold tolerance	82
qCTGERM4-5	*JAPONICA*	*qCTS4-2*, L48 [7)], *qLTG-4*	4	30,772,388-32,650,528	31,428,589	cold tolerance	31, 13, 82
	temperate japonica	*qCTS4-2*	4	30,772,388-32,650,528	31,428,589	cold tolerance	31, 13, 82
qCTGERM5-1	*tropical japonica*	*qCTS5*	5	19,189,417-22,051,412	21,484,489	cold tolerance	48
	tropical japonica	*qCTS5*, L51[6),7)], *qLTG-5-2*[6)], *qCTS5*[6)]	5	19,189,417-22,051,412	21,736,056	cold tolerance	48, 13, 45, 46
qCTGERM6-1	*JAPONICA*	*qCTS6-1*[6)]	6	5,425,408-5,425,631	6,014,941	cold tolerance	1, 55, 82, 47
	JAPONICA	*COLD4*	6	4,303,092-9,309,108	6,014,941	cold tolerance	47
qCTGERM6-2	*INDICA*	*qCTS6-2*	6	6,229,689	6,229,689	cold tolerance	32
qCTGERM7-1	*tropical japonica*	L72[6),7)], *qCTS7* [6)] *qPSST-7* [6)],	7	10,627,289	10,627,289	cold tolerance	49, 46
qCTGERM7-4	*ALL*	*qCTS7(2)*	7	17,470,108-22,550,963	20,134,258	cold tolerance	48

	ALL	*qCTS7-2*	7	17,876,290	17876290	cold tolerance	32
	JAPONICA	*qCTS7(2)*	7	17,470,108-22,550,963	20,000,802	cold tolerance	48
qCTGERM7-5	*tropical japonica*	L79[7)]	7	27,844,441	27,844,441	BMR	13
qCTGERM8-1	*ALL*	*qCTF8*	8	8,497,626- 10,798,150	10,476,180	cold tolerance	83
	ALL	*qCTS8-3*	8	10,507,858	10,507,858	cold tolerance	32
qCTGERM10-2	*tropical japonica*	*qCTS10*, L102 [7)], *qLTG-10*	10	9,692,509-16,358,895	10,257,370	cold tolerance	82, 14, 45 13
qCTGERM10-3	*INDICA*	*qCST10*, L107[7)]	10	22,395,026	22,395,026	cold tolerance	46, 13
qCTGERM11-4	*INDICA*	*qCTS11-5*	11	17,985,707	17,985,707	cold tolerance	32
qCTGERM11-5	*temperate japonica*	*qCTS11(2)-2*	11	19,639,219-22,014,679	19,718,317	cold tolerance	48
qCTGERM12-2	*ALL*	*qCTF12*	12	23,494,445-27,466,213	26,426,057	cold tolerance	83
	INDICA	*qCTF12*	12	23,494,445-27,466,213	26,366,145	cold tolerance	83
qCTGERM12-3	*INDICA*	*qCTF12*	12	23,494,445-27,466,213	27,219,850	cold tolerance	83
**Reproductive**							
qSWTPNCT1-1	SWPan	*Pdw1-1*[6)]	1	3,509,004-3,509,959	3,451,811	panicle weight	52, 58, 27
qSWTCT1-2	SWPlt	*qSW1-1*	1	41,967,890-42,955,596	41,861,649	seed weight	50
qSWTPNCT2-1	SWPan	*PNWT2*	2	18,225,635-27,038,920	18,745,789	panicle weight	84, 60
	SWPan	*GWP2*[6)]	2	18,225,635-27,038,920	18,756,457	grain yield per panicle	54
qFERCT2	Percent sterility	*nfg2*	2	17,484,665-21,766,231	19,425,953	filled grain number	62
	Percent sterility	*qLTG2*	2	19,375,071	19,375,071	low temperature germinability	31
qSWTPNCT2-2	SWPan	*yld2.1*	2	28,688,829-35,011,706	28,665,713	grain yield	53
	SWPan	*qnob-5*	2	26,242,374-28,634,075	28,665,713	no. branches per panicle	58
qSWTPNCT5	SWPan	*qCTB5*[6)]	5	27,342,022-27,342,124	27,195,071	cold tolerance	6
qSWTPNCT6-1	SWPan	*qCTS6-1*[6)]	6	5,425,408-5,425,631	5,389,785	cold tolerance	1, 55
qFERCT6-4	Percent sterility	L63	6	16,769,687	16,769,687	ELSC1	13
qSWTCT6	SWPlt	L66[7)]	6	24,368,838	24,368,838	RLC	13
qSWTPNCT6-2	SWPan	*GWP6*	6	22,862,400-26,253,308	24,812,032	grain yield per panicle	54, 58
	SWPan	*qple-4*	6	24,036,500-28,600,180	24,812,032	panicle length	58
qSWTPNCT7-1	SWPan	*qLTG7*	7	1,905,597	1,905,597	low temperature germinability	31
qFERCT7	Percent sterility	*gp7*	7	7,232,181-16,874,331	14,316,346	filled grain number	59, 61
	Percent sterility	*PSS7*	7	12,785,560-19,504,131	14,316,346	spikelet sterility	54
qSWTCT8-4	SWPlt	L87[7)]	8	26,645,812	26,645,812	ELSR1	13

qFERCT12	Percent sterility	L125[7)]	12	7,312,826	7,312,826	ELSR2	13
qSWTCT12	SWPlt	*gw12.1*	12	26,107,904-26,954,947	26,116,605	100-grain weight	57
		qCTSSR12-1	12	26,107,904-26,108,004	26,116,605	cold tolerance	51, 14

[1)] "SWPan" is the mean seed weight per panicle and "SWPlt" is the mean seed weight per plant.

[2)] Trait descriptions (13) are BMR (fresh versus dry biomass ratio after natural chilling stress), ELC2 (electrolyte leakage after natural chilling stress for 7 d), ELSC1 (electrolyte leakage after cold shock for 1 d), ELSR1 (ratio of electrolyte leakage under 1 d cold shock stress to normal condition), ELSR2 (ratio of electrolyte leakage under 3 d cold shock stress to normal condition), and RLC [resistance level (score) under natural chilling stress].

[3)] Most of these QTLs also are identified in GRAMENE (40).

[4)] QTL positions listed in Wang et al. (32) based on the MSU6 annotation, were converted to the MSU7 annotation to identify overlapping QTLs.

[5)] References not cited in the manuscript are listed below.

82. Kashiwagi T, Ishimaru K. Identification and Functional Analysis of a Locus for Improvement of Lodging Resistance in Rice. Plant Physiol. 2004;134: 676–683. doi:10.1104/pp.103.029355

83. Shinada H, Iwata N, Sato T, Fujino K. QTL pyramiding for improving of cold tolerance at fertilization stage in rice. Breed Sci. 2014; 63(5): 483-488

84. Obara M, Kajiura M, Fukuta Y, Yano M, Hayashi M, Yamaya T, et al. Mapping of QTLs associated with cytosolic glutamine synthetase and NADH-glutamate synthase in rice (*Oryza sativa* L.). J Exp Bot. 2001; 52(359): 1209-1217

[6)] SNP near to QTL.

[7)] "L" is for Locus as reported by Lv et al. (13).
