# Supplementary material for: Genetic architecture of cold tolerance in rice (Oryza sativa) determined through high resolution genome-wide analysis
Source: PLoS One. 2017 Mar 10;12(3):e0172133. doi: 10.1371/journal.pone.0172133 (PMC5345765; doi:10.1371/journal.pone.0172133)
Supplement: S5 Table — These candidate genes were verified as part of cold tolerance studies in other plant species. (PDF) [file pone.0172133.s005.pdf]

S5 Table. Summary of candidate genes co-located with SNPs identified in this study as associated with cold tolerance at the germination and reproductive stages. These candidate genes were verified as part of cold tolerance studies in other plant species.

QTL	Population group /Trait[1)]	Gene	Chr.	Position (Mb)	Distance from targeted region (bp)	Description
**Germination**						
qCTGERM1-1	*ALL*	LOC_Os01g02750	1	0.954-0.956	0	protein kinase domain containing protein, expressed
qCTGERM1-2	*JAPONICA*	LOC_Os01g07980	1	3.855-3.856	883	ankyrin, putative, expressed
qCTGERM1-3	*ALL*	LOC_Os01g14630	1	8.191-8.192	549	polyprenyl synthetase, putative, expressed
qCTGERM1-8	*ALL*	LOC_Os01g71830	1	41.603-41.604	0	glycosyl hydrolases family 17, putative, expressed
qCTGERM1-9	*JAPONICA*	LOC_Os01g74470	1	43.125-43.134	0	ABC transporter, ATP-binding protein, putative, expressed
qCTGERM2-2	*ALL*	LOC_Os02g12540	2	6.556-6.558	0	cytochrome P450, putative, expressed
	INDICA	LOC_Os02g12540	2	6.556-6.558	0	cytochrome P450, putative, expressed
qCTGERM2-4	*ALL*	LOC_Os02g36150	2	21.788-21.791	0	cytochrome P450, putative, expressed
	INDICA	LOC_Os02g36140	2	21.765-21.773	65,266	terpene synthase, putative, expressed
	JAPONICA	LOC_Os02g36150	2	21.788-21.791	6,740	cytochrome P450, putative, expressed
	temperate japonica	LOC_Os02g36150	2	21.788-21.791	0	cytochrome P450, putative, expressed
	INDICA	LOC_Os02g36210	2	21.841-21.847	2,375	ent-kaurene synthase, chloroplast precursor, putative, expressed
qCTGERM4-1	*temperate japonica*	LOC_Os04g02410	4	0.8681-0.8687	1,767	expressed protein
	JAPONICA	LOC_Os04g02450	4	0.8884-0.8907	22,058	rust-resistance protein Lr21, putative, expressed
	ALL	LOC_Os04g02850	4	1.110-1.113	3,391	pentatricopeptide, putative, expressed
qCTGERM4-3	*INDICA*	LOC_Os04g45950	4	27.217-27.214	0	amino acid permease family protein, putative, expressed
qCTGERM4-4	*JAPONICA*	LOC_Os04g52800	4	31.434-31.435	508	expressed protein
	temperate japonica	LOC_Os04g52920	4	31.524-31.527	443	remorin family protein, putative, expressed
qCTGERM5-1	*tropical japonica*	LOC_Os05g36240	5	21.482-21.485	0	expressed protein
qCTGERM6-1	*JAPONICA*	LOC_Os06g11410	6	6.019-6.021	4,656	cyclin, putative, expressed
qCTGERM6-2	*ALL*	LOC_Os06g11650	6	6.177-6.179	166	phosphate-induced protein 1 conserved region domain containing protein, expressed
qCTGERM7-4	*ALL*	LOC_Os07g33690	7	20.129-20.135	0	NBS-LRR type disease resistance protein Hom-F, putative, expressed
	JAPONICA	LOC_Os07g33480	7	20.012-20.015	11,932	cytochrome P450 domain containing protein, expressed
	JAPONICA	LOC_Os07g33600	7	20.067-20.068	1,234	hydroxylase, putative, expressed

	JAPONICA	LOC_Os07g33670	7	20.115-20.123	300	patatin-like phospholipase family protein, expressed
qCTGERM8-1	*ALL*	LOC_Os08g17080	8	10.453-10.458	18,135	PPR repeat domain containing protein, putative, expressed
qCTGERM8-2	*JAPONICA*	LOC_Os08g20420	8	12.266-12.269	1,274	MGD2, putative, expressed
qCTGERM9-1	*INDICA*	LOC_Os09g17670	9	10.804-10.804	1,310	expressed protein
qCTGERM10-2	*tropical japonica*	LOC_Os10g20390	10	10.249-10.252	4,808	MATE efflux family protein, putative, expressed
qCTGERM11-3	*JAPONICA*	LOC_Os11g28270	11	16.249-16.258	1,004	zinc finger C-x8-C-x5-C-x3-H type family protein, expressed
qCTGERM11-4	*INDICA*	LOC_Os11g29970	11	17.411-17.417	0	NB-ARC domain containing protein, expressed
qCTGERM11-5	*JAPONICA*	LOC_Os11g33330	11	19.709-19.718	0	peptidase, M24 family protein, putative, expressed
	temperate japonica	LOC_Os11g33330	11	19.709-19.718	0	peptidase, M24 family protein, putative, expressed
qCTGERM12-2	*ALL*	LOC_Os12g42540	12	26.424-26.425	933	zinc finger, C3HC4 type domain containing protein, expressed
	INDICA	LOC_Os12g42420	12	26.363-26.365	441	DNA binding protein, putative, expressed
	INDICA	LOC_Os12g42550	12	26.425-26.430	0	methyl-CpG binding domain containing protein, putative, expressed
qCTGERM12-3	*INDICA*	LOC_Os12g43840	12	27.195-27.281	791	ankyrin repeat domain-containing protein, putative, expressed
**Reproductive**						
qSWTPNCT1-1	SWPan	LOC_Os01g07310	1	3.446-3.450	1,589	transporter-related, putative, expressed
qSWTCT1-1	SWPlt	LOC_Os01g72190	1	41.861-41.863	87	glutathione S-transferase, putative, expressed
qSWTCT1-2	SWPlt	LOC_Os01g72250	1	41.892-41.896	0	uridine 5-monophosphate synthase, putative, expressed
qSWTPNCT1-3	SWPan	LOC_Os01g74300	1	43.046-43.047	389	metallothionein, putative, expressed
qSWTPNCT2-1	SWPan	LOC_Os02g31280	2	18.746-18.747	12,578	expressed protein
qFERCT2	Percent sterility	LOC_Os02g32700	2	19.423-19.423	2,003	autophagy-related protein, putative, expressed
qSWTPNCT2-2	SWPan	LOC_Os02g47744	2	29.190-29.195	2,847	MYB family transcription factor, putative, expressed
qSWTPNCT5	SWPan	LOC_Os05g47470	5	27.198-27.199	3,283	VIP1 protein, putative, expressed
qFERCT6-3	Percent sterility	LOC_Os06g25250	6	14.770-14.773	123	endoribonuclease Dicer, putative, expressed
qSWTCT6	SWPlt	LOC_Os06g41300	6	24.712-24.712	1,378	expressed gene
qSWTPNCT6-1	SWPan	LOC_Os06g10470	6	5.388.-5.389	771	ZOS6-02 - C2H2 zinc finger protein, expressed
qFERCT7	Percent sterility	LOC_Os07g25060	7	14.303-14.303	12,472	thionin-like peptide, putative, expressed
qSWTCT7	SWPlt	LOC_Os07g38620	7	23.194-23.199	0	pyridoxamine 5'-phosphate oxidase family protein, putative, expressed
qSWTCT8-1	SWPlt	LOC_Os08g02094	8	0.686-0.693	0	GDSL-like lipase/acylhydrolase, putative, expressed
qSWTPNCT8	SWPan	LOC_Os08g05510	8	2.934-2.940	2,060	MYB family transcription factor, putative, expressed

qSWTCT8-5	SWPlt	LOC_Os08g44560	8	28.014-28.020	0	expressed gene
qSWTCT8-2	SWPlt	LOC_Os08g06710	8	3.763-3.764	0	OsFBDUF42 - F-box and DUF domain containing protein, expressed
qSWTCT8-3	SWPlt	LOC_Os08g10608	8	6.243-6.247	962	ribosomal protein S17, putative, expressed
qSWTCT12	SWPlt	LOC_Os12g42130	12	26.117-26.120	2,595	MATE efflux family protein, putative, expressed

[1)] "SWPan" is the mean seed weight per panicle and "SWPlt" is the mean seed weight per plant.
