# Supplementary material for: Genetic architecture of cold tolerance in rice (Oryza sativa) determined through high resolution genome-wide analysis
Source: PLoS One. 2017 Mar 10;12(3):e0172133. doi: 10.1371/journal.pone.0172133 (PMC5345765; doi:10.1371/journal.pone.0172133)
Supplement: S6 Table — Regions where position of significant SNPs (Table 2) overlapped with the candidate genes (S5 Table) based on the germination index across the population groups. (PDF) [file pone.0172133.s006.pdf]

S6 Table. Overlap between candidate genes and significant SNPs. Regions where position of significant SNPs (Table 2) overlapped with the candidate genes (S5 Table) based on the germination index across the population groups.

Chromo-some	Position of segment (Mb)	SNP position (Mb)	Gene	Population group(s)
1	0.939-1.066	0.956	LOC_Os01g02750	*ALL*
1	3.712-3.995	3.856	LOC_Os01g07980	*JAPONICA*
1	8.161-8.228	8.19	LOC_Os01g14630	*ALL*
1	11.338-11.881	11.338		*tropical japonica*
1	12.710-12.725	12.714		*temperate japonica*
1	22.892-22.917	22.917		*JAPONICA*
1	27.060-27.169	27.06		*JAPONICA*
1	41.518-41.786	41.603	LOC_Os01g71830	*ALL, temperate japonica*
1	43.039-43.223	43.128	LOC_Os01g74470	*JAPONICA*
2	1.068-1.655	1.329		*ALL*
2	6.037-6.624	6.557	LOC_Os02g12540	*ALL, INDICA*
2	8.487-8.905	8.885		*ALL*
2	21.772-21.872	21.797	LOC_Os02g36140	*ALL, JAPONICA, temperate japonica*
2	22.650-22.661	22.661		*ALL*
4	0.866-1.116	0.866	LOC_Os04g02450	*JAPONICA, temperate japonica*
4	1.103-1.648	1.116	LOC_Os04g02850	*ALL*
4	27.214-27.766	27.214	LOC_Os04g45950	*INDICA*
4	31.047-32.230	31.159	LOC_Os04g52920	*ALL, JAPONICA, temperate japonica*
5	21.435-21.485	21.484	LOC_Os05g36240	*tropical japonica*
6	6.142-6.226	6.178	LOC_Os06g11650	*ALL, JAPONICA, INDICA*
7	10.464-10.647	10.525		*tropical japonica*
7	13.211-13.359	13.359		*tropical japonica*
7	27.860-27.947	27.947		*tropical japonica*
7	17.133-17.353	17.343		*ALL*

7	19.794-20.262	20.134	LOC_Os07g33600	*ALL, JAPONICA*
7	20.115-20.123	20.123	LOC_Os07g33670	*JAPONICA*
8	10.438-10.903	10.476	LOC_Os08g17080	*ALL*
8	12.270-12.345	12.27	LOC_Os08g20420	*JAPONICA*
9	10.760-10.911	10.806	LOC_Os09g17670	*INDICA*
10	1.403-1.526	1.52		*ALL*
10	3.009-3.111	3.009		*ALL*
10	10.165-10.335	10.257		*tropical japonica*
11	1.6248-1.624	1.624		*temperate japonica*
11	5.590-5.741	5.59		*ALL*
11	17.303-17.425	17.413	LOC_Os11g29970	*INDICA*
11	19.676-19.770	19.718		*JAPONICA, temperate japonica*
12	24.896-24.904	24.904		*JAPONICA*
12	26.314-26.988	26.426		*ALL, JAPONICA, INDICA*
12	27.195-27.281	27.219		*INDICA*
