# Supplementary material for: Genetic architecture of cold tolerance in rice (Oryza sativa) determined through high resolution genome-wide analysis
Source: PLoS One. 2017 Mar 10;12(3):e0172133. doi: 10.1371/journal.pone.0172133 (PMC5345765; doi:10.1371/journal.pone.0172133)
Supplement: S1 Fig — Distribution of the “Germination Index” calculated as percentage of seeds with coleoptiles >5 mm in cold treatment to percentage of seeds with coleoptiles >5 mm in warm treatment, across ALL RDP1 accessions is the sum of the gray and hash bars. The hash bars indicate the frequency distribution for the subset of A) INDICA (indica, aus, admix-INDICA) accessions and B) JAPONICA (tropical japonica, temperate japonica, admix-JAPONICA) accessions. Quantile box-and-whisker plots show the distribution observed values and the mean for the INDICA and JAPONICA accessions. The cold tolerant check, Quilla66304 is shown. (Table 1 lists additional statistics.). (PDF) [file pone.0172133.s007.pdf]

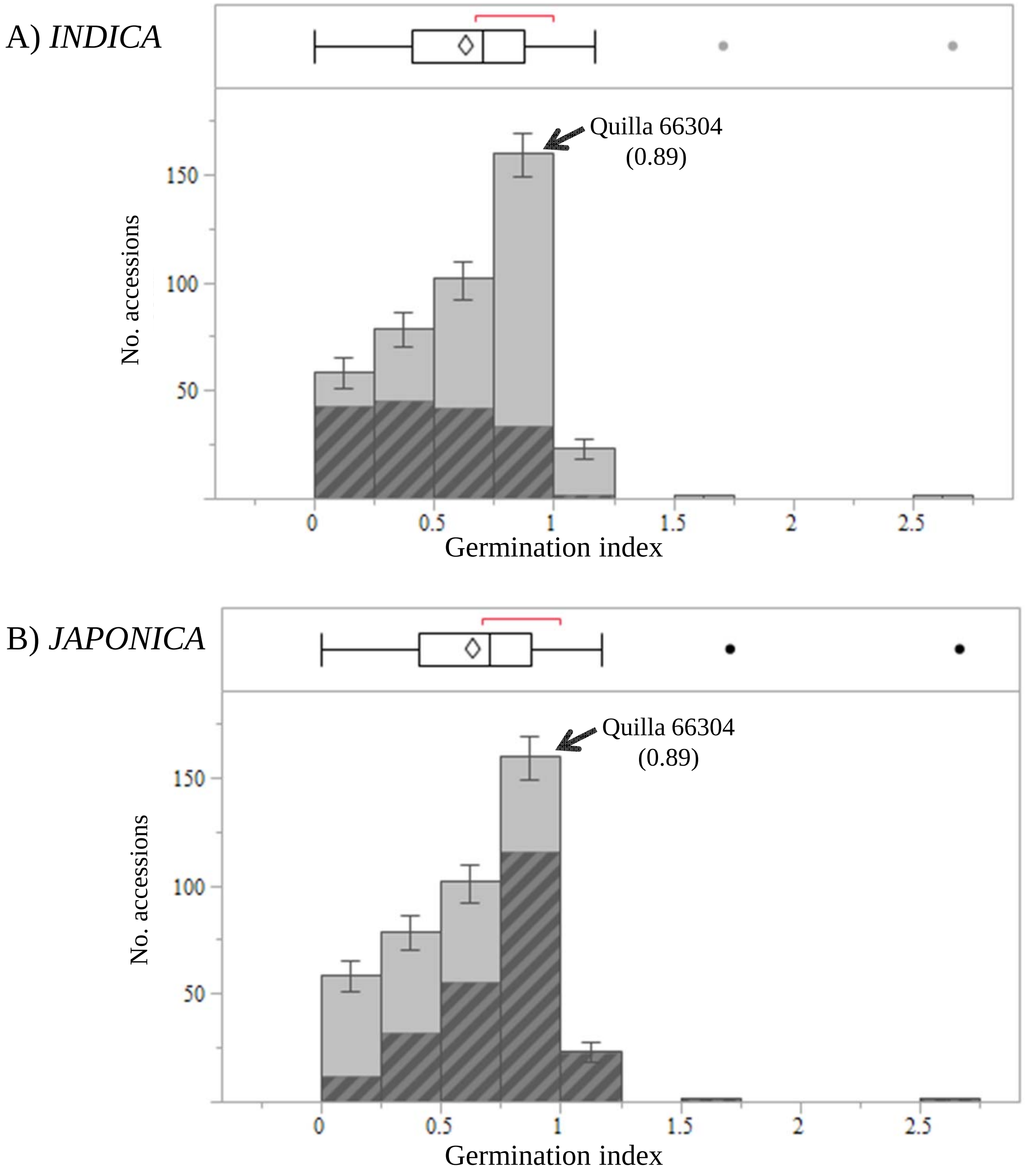

S1 Fig. Frequency distribution of the adjusted cold germination ratio for the RDP1 *O. sativa* accessions. Distribution of the “Germination Index” calculated as percentage of seeds with coleoptiles > 5mm in cold treatment to percentage of seeds with coleoptiles > 5 mm in warm treatment, across *ALL* RDP1 accessions is the sum of the gray and hash bars. The hash bars indicate the frequency distribution for the subset of A) *INDICA* (*indica*, *aus*, admix-*INDICA*) accessions and B) *JAPONICA* (*tropical japonica*, *temperate japonica*, admix-*JAPONICA*) accessions. Quantile box-and-whisker plots show the distribution observed values and the mean for the *INDICA* and *JAPONICA* accessions. The cold tolerant check, Quilla66304 is shown. (Table 1 lists additional statistics.)
