# Supplementary material for: Genetic architecture of cold tolerance in rice (Oryza sativa) determined through high resolution genome-wide analysis
Source: PLoS One. 2017 Mar 10;12(3):e0172133. doi: 10.1371/journal.pone.0172133 (PMC5345765; doi:10.1371/journal.pone.0172133)
Supplement: S2 Fig — The distribution of the mean percent sterility, [classified as category 1 (0 to 5% sterility), 2 (6 to 12%), 3 (13 to 18%), 4 (19 to 40%), 5 (41 to 60%), 6 (61 to 90%) and 7 (91 to 100%)], mean seed weight per panicle and mean seed weight per plant as a measure of cold tolerance at booting (panicle emergence) in rice. Quantile box-and-whisker plots show the distribution of observed values and the mean for each trait. The value for cold tolerant check, Mustakilik, is shown. (Table 1 lists additional statistics.). (PDF) [file pone.0172133.s008.pdf]

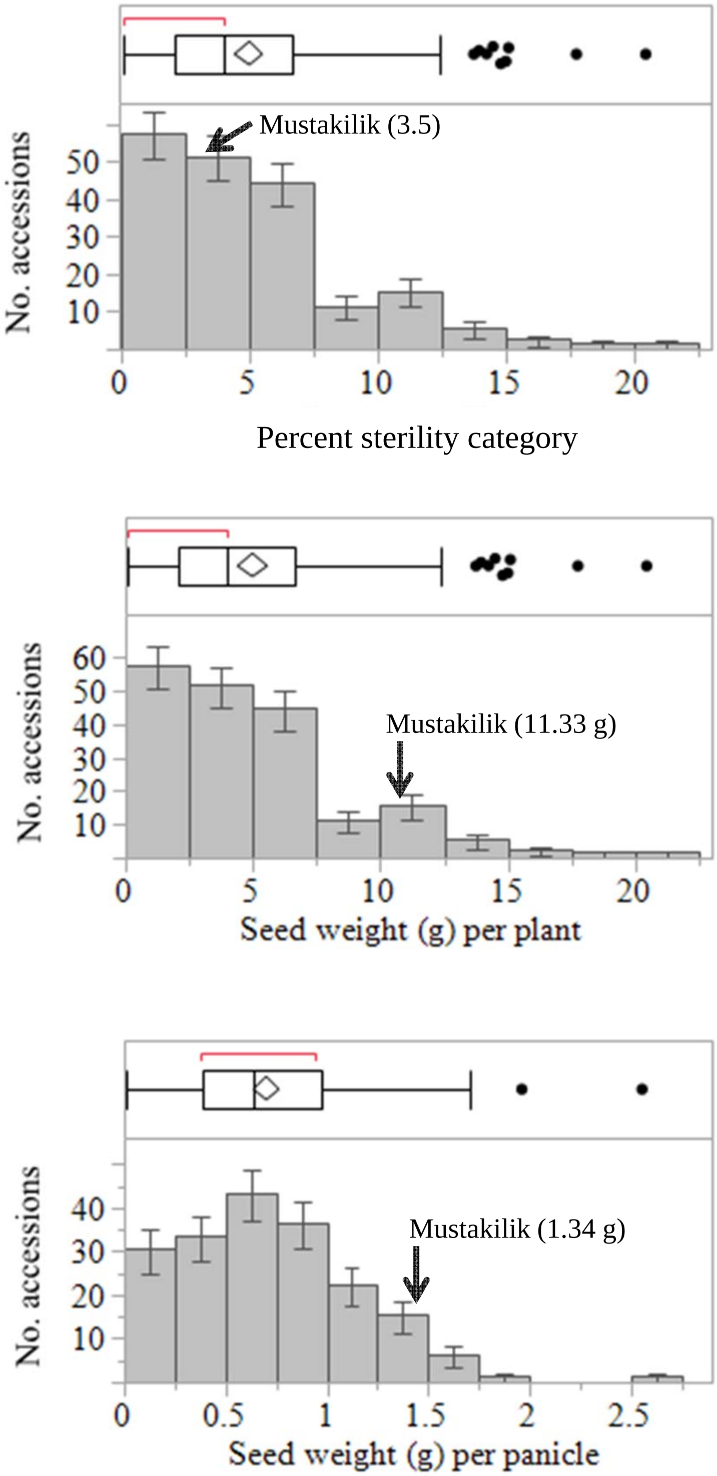

S2 Fig. Distribution of the 189 *JAPONICA* RDP1 accessions for three reproductive traits as a measure of cold tolerance at booting. The distribution of the mean percent sterility [classified as category 1 (0-5% sterility), 2 (6-12%), 3 (13-18%), 4 (19-40%), 5 (41 to 60%), 6 (61 to 90%) and 7 (91 to 100%)], mean seed weight per panicle and mean seed weight per plant as a measure of cold tolerance at booting (panicle emergence) in rice. Quantile box-and-whisker plots show the distribution of observed values and the mean for each trait. The value for cold tolerant check, Mustakilik, shown. (Table 1 lists additional statistics.)
