# Supplementary material for: Genetic architecture of cold tolerance in rice (Oryza sativa) determined through high resolution genome-wide analysis
Source: PLoS One. 2017 Mar 10;12(3):e0172133. doi: 10.1371/journal.pone.0172133 (PMC5345765; doi:10.1371/journal.pone.0172133)
Supplement: S3 Fig — The y-axis is the observed number and magnitude of observed p-value (-10 log) displays associations between SNPs and cold tolerance and the x-axis is the expected p-value (-10 log) under the null hypothesis (there is no association between SNPs and cold tolerance). The λ (lambda) is the genomic inflation factor. (PDF) [file pone.0172133.s009.pdf]

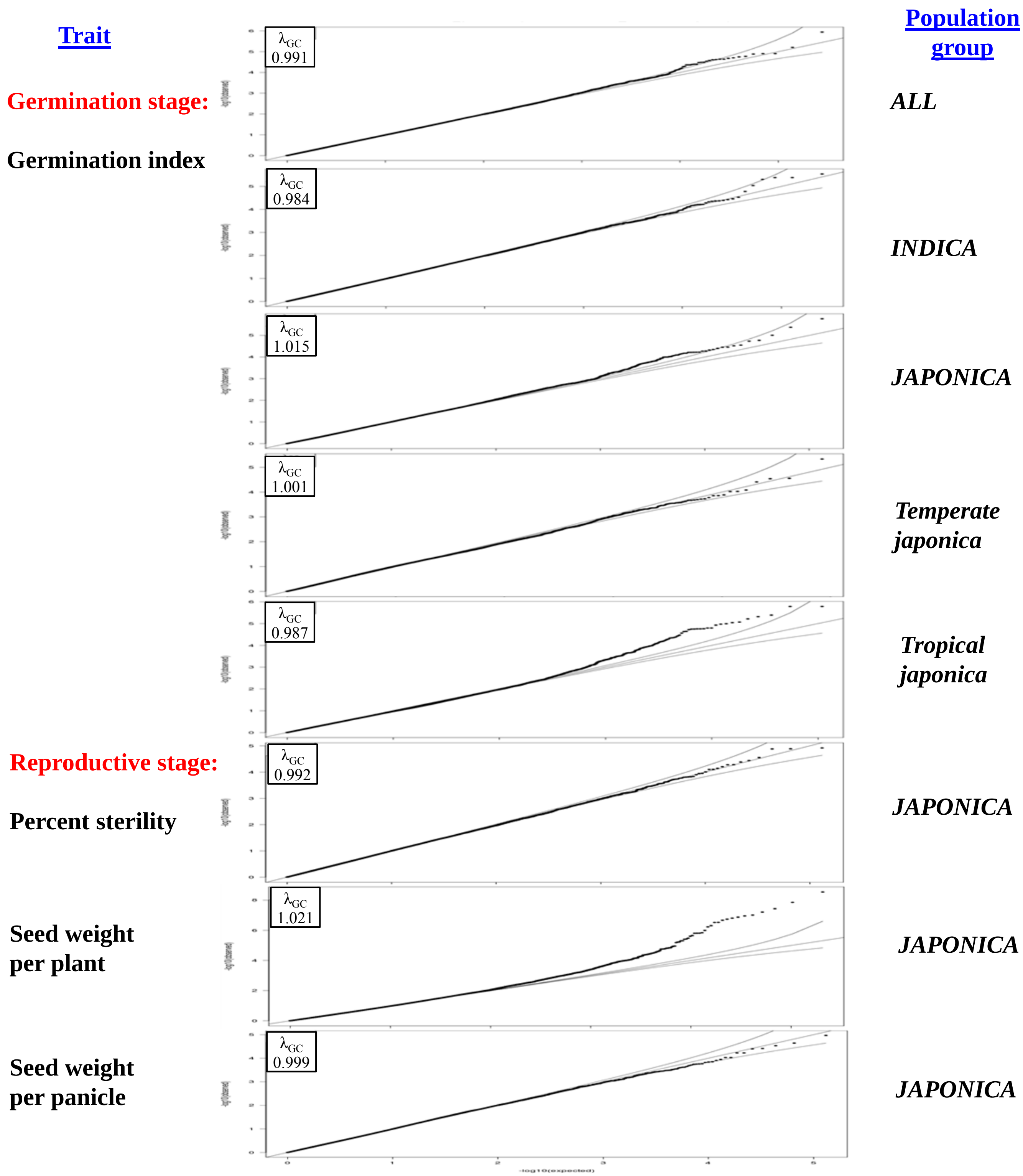

S3 Fig. Quantile-Quantile plots for four cold tolerance traits measured at germination and at the reproductive stages. The *y*-axis is the observed number and magnitude of observed *p*-value (-10 log) displays associations between SNPs and cold tolerance and the *x*-axis is the expected *p*-value (-10 log) under the null hypothesis (there is no association between SNPs and cold tolerance). The λ (lambda) is the genomic inflation factor.
